# Hutterite‐type cataract maps to chromosome 6p21.32‐p21.31, cosegregates with a homozygous mutation in *LEMD2*, and is associated with sudden cardiac death

**DOI:** 10.1002/mgg3.181

**Published:** 2015-11-14

**Authors:** Philip M. Boone, Bo Yuan, Shen Gu, Zhiwei Ma, Tomasz Gambin, Claudia Gonzaga‐Jauregui, Mahim Jain, Todd J. Murdock, Janson J. White, Shalini N. Jhangiani, Kimberly Walker, Qiaoyan Wang, Donna M. Muzny, Richard A. Gibbs, J. Fielding Hejtmancik, James R. Lupski, Jennifer E. Posey, Richard A. Lewis

**Affiliations:** ^1^ Department of Molecular and Human GeneticsBaylor College of MedicineHoustonTexas; ^2^ Ophthalmic Genetics and Visual Function BranchNational Eye InstituteRockvilleMaryland; ^3^ Rocky Mountain Eye CenterMissoulaMontana; ^4^ Human Genome Sequencing CenterBaylor College of MedicineHoustonTexas; ^5^ Department of PediatricsBaylor College of MedicineHoustonTexas; ^6^ Texas Children's HospitalHoustonTexas; ^7^ Department of OphthalmologyBaylor College of MedicineHoustonTexas; ^8^ Department of MedicineBaylor College of MedicineHoustonTexas

**Keywords:** Cataract, Hutterite, LEM domain, *LEMD2*, *MUC21*, recessive, sudden death

## Abstract

**Background:**

Juvenile‐onset cataracts are known among the Hutterites of North America. Despite being identified over 30 years ago, this autosomal recessive condition has not been mapped, and the disease gene is unknown.

**Methods:**

We performed whole exome sequencing of three Hutterite‐type cataract trios and follow‐up genotyping and mapping in four extended kindreds.

**Results:**

Trio exomes enabled genome‐wide autozygosity mapping, which localized the disease gene to a 9.5‐Mb region on chromosome 6p. This region contained two candidate variants, *LEMD2* c.T38G and *MUC21* c.665delC. Extended pedigrees recruited for variant genotyping revealed multiple additional relatives with juvenile‐onset cataract, as well as six deceased relatives with both cataracts and sudden cardiac death. The candidate variants were genotyped in 84 family members, including 17 with cataracts; only the variant in *LEMD2* cosegregated with cataracts (LOD = 9.62). SNP‐based fine mapping within the 9.5 Mb linked region supported this finding by refining the cataract locus to a 0.5‐ to 2.9‐Mb subregion (6p21.32‐p21.31) containing *LEMD2* but not *MUC21*. *LEMD2* is expressed in mouse and human lenses and encodes a LEM domain‐containing protein; the c.T38G missense mutation is predicted to mutate a highly conserved residue within this domain (p.Leu13Arg).

**Conclusion:**

We performed a genetic and genomic study of Hutterite‐type cataract and found evidence for an association of this phenotype with sudden cardiac death. Using combined genetic and genomic approaches, we mapped cataracts to a small portion of chromosome 6 and propose that they result from a homozygous missense mutation in *LEMD2*.

## Introduction

Juvenile cataracts develop in childhood in the crystalline lens that was clear at birth (Francis and Moore 2004). In the absence of trauma, and particularly when family history, bilaterality, or abnormalities of other organs exist, a genetic etiology for cataracts may be suspected. For example, several inborn errors of galactose metabolism can cause juvenile cataracts, either in isolation (galactokinase deficiency, MIM #230200) or with an overall metabolic phenotype (classical galactosemia, MIM #230400; and galactose epimerase deficiency, MIM #230350). Numerous genetic forms of cataract are known, and both isolated (nonsyndromic) and syndromic forms have been reviewed (Francis et al. 2000; Francis and Moore 2004; Shiels et al. 2010; Shiels and Hejtmancik 2013).

Lowry and colleagues (Shokeir and Lowry 1985; Pearce et al. 1987) described nonsyndromic juvenile cataracts in a single large Hutterite family. Hutterites are an Anabaptist religious isolate living in the northwestern United States and southwestern Canada. Because of both their geographic and social isolation and their common ancestry, the coefficient of inbreeding of Hutterite unions is high (Pearce et al. 1987). The high coefficient of inbreeding and the pattern of trait segregation led Shokeir and Lowry (1985) to hypothesize autosomal recessive inheritance for the cataract phenotype. In those several reports, age of onset ranged from infancy through school age, mostly between 3 and 7 years. Cataracts were cortical and described as “white” or “opaque,” and the time to progression to a mature cataract was rapid (1–3 months). In the two original reports, no other associated eye, body, or cognitive abnormalities were described, although all affected individuals were young at the time of publication. No trauma, infection, or metabolic abnormalities (urinary reducing substances, decreased erythrocyte galactokinase activity) were found. Contemporary surgery was fully corrective, although secondary membrane formation in some individuals necessitated later needling. The phenotype of each individual is described in Table S1.

The aforementioned family belongs to the Lehrerleut group of Hutterites (Hostetler and Huntington 1980). Pearce et al. (1987) estimated the carrier frequency for juvenile cataracts in Lehrerleut Hutterites to be high (~0.12); thus, additional cases were anticipated. Yet, no further information about this phenotype, now termed “Hutterite‐type cataract” (OMIM %212500), has been published in the last three decades. Of particular importance, no genetic studies have been done to map the phenotype to a chromosomal location, and the causal mutation and disease gene remain unknown.

We performed whole exome sequencing of DNA from three Hutterite individuals with juvenile cataracts and their parents. We used exome data to perform genome‐wide autozygosity mapping, which localized the disease locus to a 9.5‐Mb region of chromosome 6p containing two candidate variants (*LEMD2* c.T38G and *MUC21* c.665delC). While the *LEMD2* variant cosegregated with cataracts in 84 family members (including 17 affected individuals), the *MUC21* variant did not. SNP‐based fine mapping within the 9.5 Mb linked region confirmed this finding by refining the locus to a 0.5‐ to 2.9‐Mb subregion (6p21.32‐p21.31) containing *LEMD2* but not *MUC21*. These data suggest that *LEMD2* is the disease gene for Hutterite‐type cataract. Intriguingly, we observed that the most common cause of death in individuals with cataracts is sudden cardiac death at an early age, suggesting an association of this phenotype with Hutterite‐type cataracts.

## Methods

### Ethical Compliance

Subjects were recruited by the Baylor–Hopkins Center for Mendelian Genomics (http://www.mendelian.org/) and were consented under a protocol approved by the Institutional Review Board for Human Subjects Research of Baylor College of Medicine (H‐29697). The research was compliant with the US Health Insurance Portability and Accountability Act and the Declaration of Helsinki.

### Subjects

Subjects consisted of 84 Lehrerleut Hutterite individuals from the northwestern United States and southwestern Canada. All individuals with juvenile cataracts have been examined by and/or undergone cataract surgery by an ophthalmologist (T.J.M., R.A.L.).

### Whole exome sequencing (WES)

DNA for exome sequencing was extracted from whole blood with the Gentra Puregene Blood Kit (Qiagen, Valencia, CA). DNA from remaining family members was extracted from blood as above or from saliva. Saliva was obtained with the Oragene•DNA OG‐575 Assisted Collection Kit (DNA Genotek, Kanata, ON, Canada) and extracted with the prepIT•CD2 Genomic DNA MiniPrep Kit (DNA Genotek) or the prepIT•L2P Reagent (DNA Genotek). Exome sequencing was performed at the Baylor College of Medicine Human Genome Sequencing Center (HGSC). An Illumina (San Diego, CA) paired end precapture library was constructed with 1 *μ*g of DNA according to the manufacturer's protocol (http://support.illumina.com/downloads/multiplexing_sample_prep_guide_1005361.html) with modifications described previously (https://www.hgsc.bcm.edu/content/protocols-sequencing-library-construction). Precapture libraries were pooled into either (1) 4‐plex library pools, then hybridized in solution to the HGSC‐designed Core capture reagent (52 Mb; AR‐899 samples) (NimbleGen; Madison, WI) (Bainbridge et al. 2011) or (2) 6‐plex library pools, then hybridized to the custom VCRome 2.1 capture reagent (42 Mb; AR‐900 samples) (NimbleGen) (Bainbridge et al. 2011) according to the manufacturer's protocol (http://www.nimblegen.com/products/lit/seqcap/ez/) with minor revisions. The sequencing run was performed in paired end mode on the Illumina HiSeq 2000 platform, with sequencing‐by‐synthesis reactions extended for 101 cycles from each end and an additional seven cycles for the index read. With sequencing yields of 10.07, 9.66, and 5.23 Gb in samples AR‐899‐06, AR‐899‐25, and AR‐900‐31, the samples achieved over 90% of the targeted exome bases covered to a depth of 20× or greater.

Illumina sequence analysis was performed with the HGSC Mercury analysis pipeline (https://www.hgsc.bcm.edu/software/mercury) that moves data through various analysis tools from the initial sequence generation on the instrument to annotated variant calls (SNPs and intra‐read indels) (Challis et al. 2012; Reid et al. 2014). Additional details of the HGSC exome sequencing and analysis pipelines have been described previously (Lupski et al. 2013) (https://www.hgsc.bcm.edu/software/mercury).

Filtering retained variants that were of high quality, potentially deleterious (missense, nonsense, stop loss, indels, and splice site variants ±5 bp around exons), and rare. A variant was considered appropriately rare if either (1) the variant was not present in the Human Gene Mutation Database (HGMD; http://www.hgmd.cf.ac.uk/) and the allele frequency was <1% in the NHLBI Exome Sequencing Project (ESP; http://evs.gs.washington.edu/EVS/), 1000 Genomes Project (1000GP; http://www.1000genomes.org), and Atherosclerosis Risk in Communities (ARIC; https://biolincc.nhlbi.nih.gov/studies/aric/) datasets or (2) the variant was present in the HGMD and the allele frequency was <5% in the ESP, 1000GP, and ARIC datasets.

### Absence of heterozygosity (AOH) mapping

Regions of AOH (autozygosity) were determined by a transformation of full SNP variant calls from exome sequencing with AgileVariantMapper software (Carr et al. 2013). Minimum read depth was set at 5, and the heterozygosity cutoff was 25% of reads.

### Genotyping the *MUC21* c.665delC variant

DNA (100 ng) served as PCR substrate in a 25 *μ*L volume containing 0.5 *μ*mol/L of each primer and 1X PCR Master Mix (Promega, Madison, WI). Thermocycler conditions were 95°C for 2 min; 40 cycles of 95°C for 30 sec, 57°C for 30 sec, and 72°C for 5 min; and 72°C for 5 min. PCR primers are listed in Table S2. PCR products were separated by gel electrophoresis. Gel bands were extracted with the Zymoclean Gel DNA Recovery Kit (Zymo Research, Irvine, CA), then Sanger sequenced (Baylor College of Medicine Sequencing Core, Houston, TX) with the *MUC21* F2 primer (mutation detection) or *MUC21* R2 primer (to complete the amplicon's sequence).

### Genotyping the *LEMD2* c.T38G mutation and SNPs used in mapping

PCR of the *LEMD2* variant was performed similarly to PCR of the *MUC21* variant, with the following alteration to the thermocycler protocol: 95°C  for 2 min; 40 cycles of 95°C for 30 sec, 57°C for 30 sec, and 72°C for 3 min; and 72°C for 5 min. PCR primers are listed in Table S2. PCR products were treated with ExoStar (GE Healthcare, Little Chalfont, UK), then sequenced as described above using either forward or reverse PCR primers.

### Cloning

Cloning utilized gel‐purified PCR products and the TOPO TA Cloning Kit for Sequencing (Life Technologies; Carlsbad, CA). Cloned PCR products were isolated via the QIAprep Spin Miniprep kit (Qiagen) and sequenced with M13 forward and reverse primers (Table S2).

### LOD score calculation

Two‐point linkage analysis was completed with FASTLINK software after consanguineous loops were broken (Cottingham et al. 1993). A recessive model with 100% penetrance for cataract and no phenocopies was utilized (i.e., AA, Aa, and aa with penetrances of 0.00, 0.00, and 1.00, respectively).

### Array comparative genomic hybridization (aCGH)

Array‐based copy‐number variant (CNV) analysis was performed with a genome‐wide Agilent (Santa Clara, CA) 1M probe oligonucleotide CGH array (format = G4824A; design ID = 02159; ~1 probe per 3 kb). Array CGH procedures followed manufacturer's instructions with modifications described previously (Gonzaga‐Jauregui et al. 2010). The hybridization control was gender mismatched. Array image files were processed with Agilent Feature Extraction software (version 10) based on genome version hg19, and CNVs called with Agilent Genomic Workbench software (version 7).

### CNV analysis of exome data

Whole exome sequencing data were transformed into per exon read depth (reads per thousand base pairs per million reads; RPKM). Homozygous deletions were called in all nine exome‐sequenced individuals with 4115 other samples as controls, based on the following criteria: (1) exons with 0 or a low number of reads (RPKM < 0.5) were identified; (2) common deletions (≥0.5% in the whole cohort) and low‐quality deletions (≥99% of samples did not have an RPKM > 1 in the candidate exon) were removed; (3) to fit with an autosomal recessive model, deletions were retained only if they overlapped with an AOH region (>0.5 Mb), calculated separately with WES data; (4) calls from consecutive exons were merged; (5) low‐quality samples with >10 homozygous/hemizygous deletions were removed.

### Quantitative RT‐PCR

Quantitative real‐time PCR (qRT‐PCR) was performed with SYBR Green (Qiagen) chemistry in an ABI VIIA7 System (Applied Biosystems, Warrington, UK). The *Lemd2* (mouse) and *LEMD2* (human) RT‐PCR primers (Table S2) produced amplicons of 138 and 137 bp, respectively. *Gapdh*/*GAPDH* was used as an endogenous control gene for normalization across samples. qRT‐PCR was performed in quadruplicate according to the recommendations of the manufacturer (Qiagen), and the data were analyzed by comparison of ΔΔCt.

### In silico analysis of *LEMD2* and *MUC21*

All human genomic coordinates are based on the February 2009 genome build (GRCh37/hg19) unless otherwise specified. *LEMD2* exon and base pair numbering are based on RefSeq transcript NM_181336.3. *MUC21* exon and base pair numbering are based on RefSeq transcript NM_001010909.2. Protein sequence conservation alignments across species were performed using the multiz alignments and conservation track of the UCSC genome browser (https://genome.ucsc.edu/index.html); sequence conservation alignments across LEM domain‐containing human proteins were performed with the NCBI protein BLAST tool (http://blast.ncbi.nlm.nih.gov/Blast.cgi).

## Results

### Four Hutterite pedigrees demonstrate autosomal recessive juvenile cataracts

**Figure 1:**
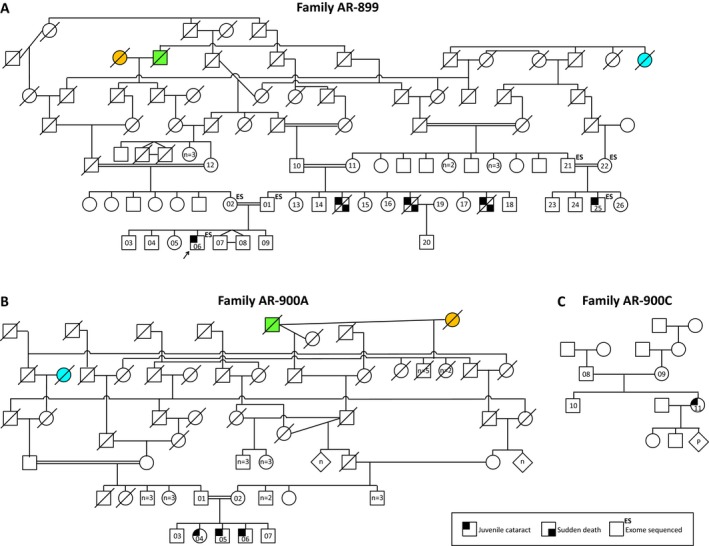
Juvenile cataracts in three Hutterite families. (A–C) Hutterite‐type cataracts in nine individuals demonstrate autosomal recessive inheritance. Consanguineous unions are known in (A) and (B). Some individuals in the ancestral generations have been removed for clarity while preserving all family relationships. Colored pedigree shapes indicate shared individuals in families AR‐899 and AR‐900A.

**Figure 2:**
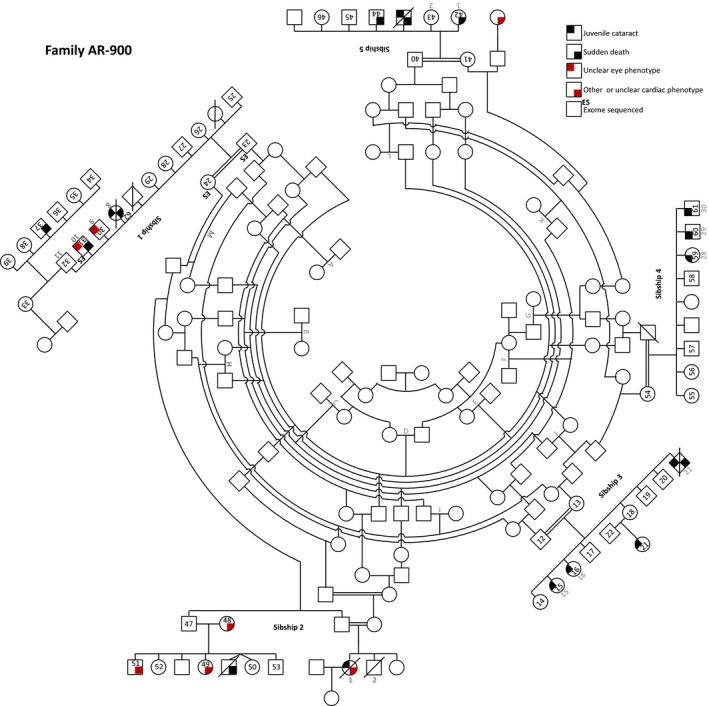
Juvenile cataracts in Hutterite family AR‐900. This consanguineous family was described previously (Shokeir and Lowry 1985; Pearce et al. 1987). The updated pedigree, including 14 individuals with cataracts, is adapted from Pearce et al. (1987) (original numbering and lettering are reproduced in gray for reference). The individual in sibship 5 with an unclear eye phenotype is described as “blind.”

The proband (AR‐899‐06), an 8‐year‐old Lehrerleut Hutterite boy, presented at age 7 with sequential cataracts in each eye occurring about 5 months apart. The cataracts were characterized by a diffuse, white haze that extended from the posterior face of the lens anteriorly to approximately the central plane of the lens (see Table S1 for additional description). Each cataract was extracted without complications. He had no other health problems. The proband's first cousin once removed (AR‐899‐25) and three paternal uncles (deceased) also had cataracts presenting in childhood (Fig. 1A). From this initial pedigree (AR‐899), we identified three additional Hutterite pedigrees (AR‐900A, AR‐900C, AR‐900) containing individuals with juvenile cataracts (Figs. 1B, 1C, 2). AR‐900 is the family reported by Lowry and colleagues in the initial reports of Hutterite‐type cataract (Shokeir and Lowry 1985; Pearce et al. 1987). Age of cataract presentation was mostly between 3 and 7 years, although one individual presented at age ~26. All pedigrees are consistent with autosomal recessive inheritance. Individuals in several pedigrees have died of sudden, apparently arrhythmogenic events in the third through fifth decades of life, and others have cardiac disease; the genetic etiology/ies of these phenotypes remain undetermined. Remarkably, of seven individuals with cataracts who have died, six experienced sudden cardiac death at an early age. Additional phenotypic data are presented as Table S1.

No prior genetic testing has been performed to establish the cause of cataracts, although some individuals in AR‐900 have had biochemical testing to exclude galactosemia/galactokinase deficiency (Shokeir and Lowry 1985; Pearce et al. 1987) (Table S1). We obtained DNA from 84 individuals, including 17 with juvenile cataracts, 22 obligate carriers, and 45 additional healthy family members. Neither extracted lens tissue nor preoperative slit lamp photobiomicrographs are available.

### Autozygosity mapping of the cataract locus to chromosome 6p

**Figure 3:**
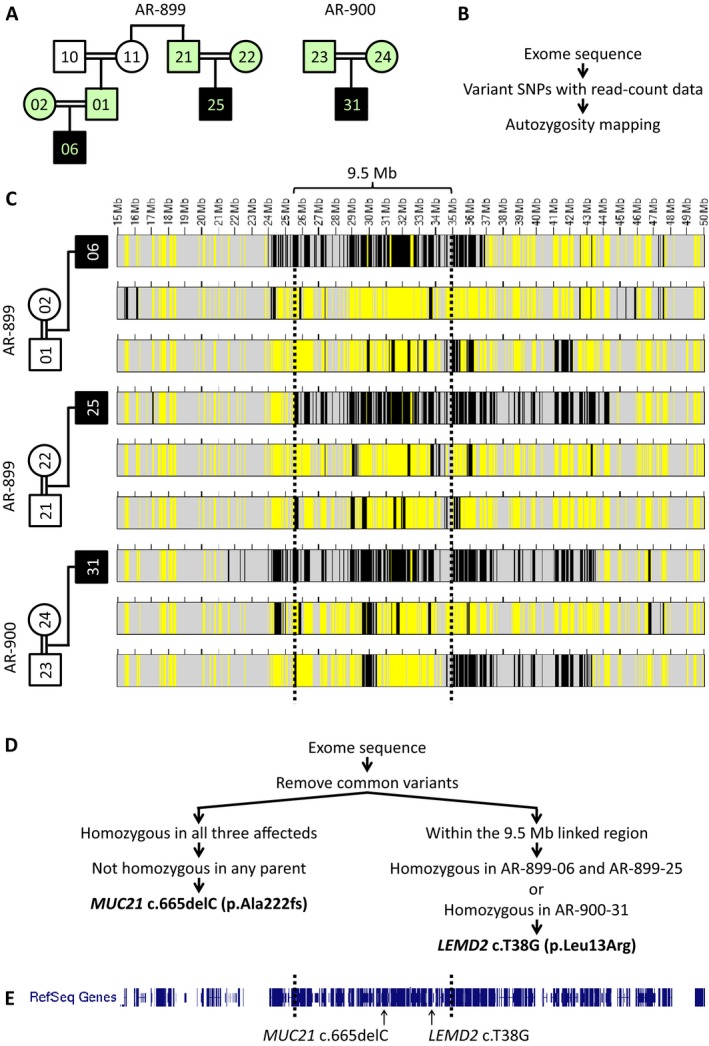
Exome sequencing of three trios enables autozygosity mapping and identification of candidate variants. (A) Exome sequencing was performed on three individuals with juvenile cataracts and their parents (mint shading). (B, C) Regions of homozygosity (black) and heterozygosity (yellow) were determined from exome variant calls using AgileVariantMapper software (Carr et al. 2013). Dotted lines demarcate a 9.5‐Mb autozygous region on chromosome 6 (6p22.2‐p21.31) shared by all three affected individuals and absent from their healthy parents, the only such region in the genome (see Fig. S1). (D) To identify candidate mutations, exome data were first filtered to include only homozygous variants unique to all three affected individuals (consistent with autosomal recessive inheritance). A single variant, *MUC21* c.665delC, satisfied these criteria. To identify variants that may have evaded sequencing in one or more samples, particularly as the AR‐899 and AR‐900 samples were captured on different platforms (AR‐899 samples were acquired several years earlier), we performed a parallel filtering scheme to identify homozygous variants within the 9.5 Mb linked region in either the AR‐899 affecteds *or* AR‐900‐31. This strategy yielded *LEMD2* c.T38G, a variant identified only in AR‐900‐31. (E) Both *MUC21* and *LEMD2* fall within the 9.5 Mb linked region on chromosome 6p.

DNA samples from three subjects with cataracts (AR‐899‐06, AR‐899‐25, and AR‐900‐31) and each of their parents (AR‐899‐01/02, AR‐899‐21/22, and AR‐900‐23/24) were subjected to whole exome sequencing (Fig. 3A). As the disease‐causing mutation for an autosomal recessive condition in a consanguineous pedigree is expected to fall within a region of autozygosity, we first used exome data to perform genome‐wide autozygosity mapping (Fig. 3B). This identified a 9.5‐Mb autozygous region on chromosome 6 (approximately chr6:25,500,000–35,000,000) shared by all three affected individuals and absent from their healthy parents (Fig. 3C). This is the sole such region in the genome (Fig. S1), indicating that Hutterite‐type cataracts map to 6p22.2‐p21.31. With the exception of *NEU1*, the disease gene for neuraminidase deficiency (OMIM #256550; may include cataracts as a minor feature), there are no known cataract genes nor mapped cataract loci within this region (UCSC Genome Browser OMIM Track, UCSC [Link]; *Cat‐Map*, Shiels et al. 2010). Of interest, this genomic interval contains the major histocompatibility complex (MHC) in which recombination is known to be suppressed (Vandiedonck and Knight 2009) (see Discussion).

### Exome sequencing identifies two candidate variants

Candidate variants were identified in two stages: First, we retained rare, homozygous alleles shared by the three affected individuals and removed variants that were also homozygous in any of the six healthy parents (Fig. 3D). Only a single variant satisfied these filtering criteria: *MUC21* c.665delC, a homozygous single‐base deletion at position chr6:30,954,617 (hg19) in the gene encoding mucin 21 (MUC21). *MUC21* is not a known disease gene, and the c.665delC variant has not been reported previously nor previously been identified in the Baylor–Hopkins Center for Mendelian Genomics (BHCMG) database. In the Exome Aggregation Consortium (ExAC; Beta version 0.2) database, which contains variants identified among 60,657 exomes, c.665delC is rare (allele frequency = 0.000166), always heterozygous, and European specific (Exome Aggregation Consortium [Link]). By conceptual translation, this is a frameshift mutation occurring at amino acid 222, predicted to add 220 aberrant amino acids to the MUC21 protein (p.Ala222Valfs*221) before premature truncation in exon 2 of 3 (Figs. S2–S3).

**Figure 4:**
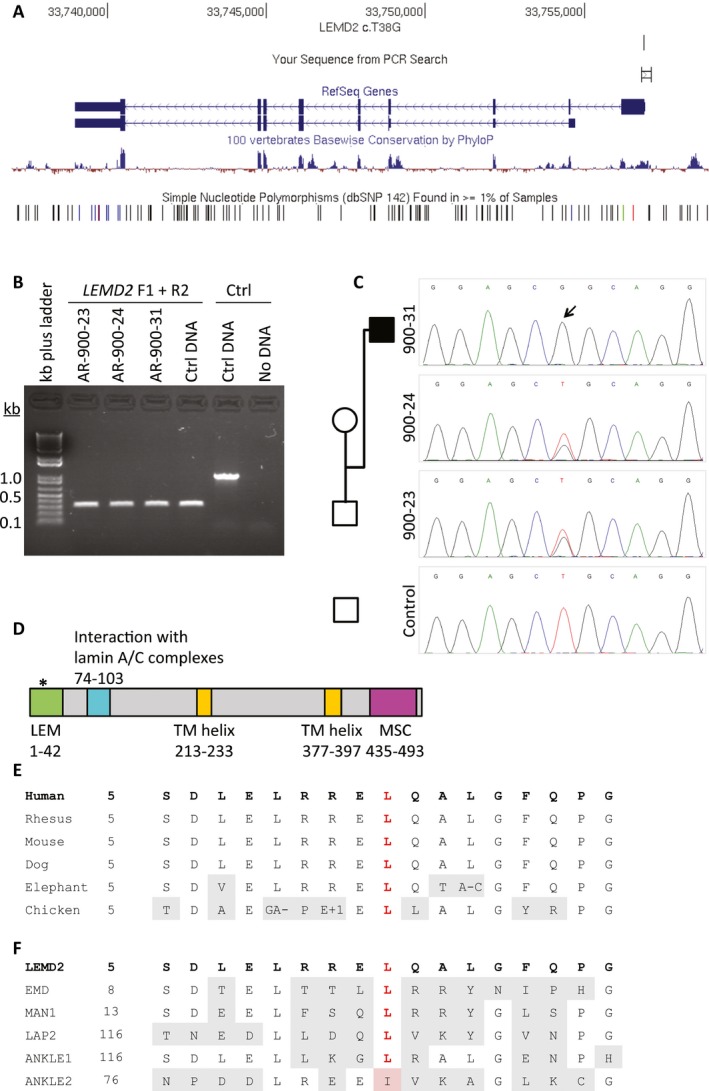
*LEMD2* c.T38G is confirmed by Sanger sequencing and is predicted to mutate the LEM domain of LEMD2. (A) *LEMD2* c.T38G falls within the first exon of the gene. The PCR amplicon for sequencing is shown (“Your Sequence from PCR Search”). (B) PCR of a trio provides substrate for sequencing. (C) Sanger sequencing of *LEMD2* c.T38G in this trio confirms its presence and cosegregation with the phenotype. Sequencing performed in reverse genomic orientation. (D) The variant is predicted to mutate the LEM domain of LEMD2. Domain information obtained from UniProt (http://www.uniprot.org/uniprot/Q8NC56) and Brachner et al. (Brachner et al. 2005). (E) The leucine residue at position 13 (red) of LEMD2 is conserved across species (adapted from UCSC Genome Browser, https://genome.ucsc.edu/index.html). (F) The leucine residue at position 13 (red) is conserved across 5 of 6 LEM domain‐containing proteins in humans, and replaced by isoleucine in ANKLE2.

A second stage was implemented to identify variants that may have evaded sequencing in one or more samples, particularly as the AR‐899 and AR‐900 samples were captured on different platforms (AR‐899 samples were acquired several years earlier). This parallel filtering scheme retained rare, homozygous variants within the 9.5 Mb linked region present in either AR‐899‐06 and AR‐899‐25 or in AR‐900‐31 (Fig. 3D). This strategy yielded *LEMD2* c.T38G, a homozygous variant at position chr6:33,756,856 (hg19) identified only in AR‐900‐31 (the affected individual sequenced with a more recent capturing technology). *LEMD2* is not a known disease gene, and the c.T38G variant has not been reported previously nor been identified previously in the BHCMG database or ExAC database. The allele was not found in any individual in the 1000 Genomes Project data (http://www.1000genomes.org) nor in the Exome Sequencing Project database (ESP; http://evs.gs.washington.edu/EVS/). By conceptual translation, this is a nonsynonymous substitution at amino acid 13, predicted to change a conserved leucine to arginine within the LEM domain of LEMD2 (p.Leu13Arg) (Fig. 4). High GC content (76%) within this exon likely contributed to reduced capture efficiency and low coverage in the AR‐899 samples (Bainbridge et al. 2010, 2011).

To determine whether whole exome sequencing was sufficient to detect variants within the linkage region, we computed the sequence read depth within and adjacent to the described linkage region, and found that 95.9% of the exons within chromosome 6:24,000,000–36,000,000 were covered by WES in at least one of the three samples at a read depth of 20× or greater (Fig. S4). While the cataract mutation is expected to be a homozygous variant on account of the overall Hutterite population history and the substantial consanguinity in the pedigrees presented here, we also performed an analysis for compound heterozygous variants similar to our first stage analysis above. No rare, potentially compound heterozygous variants are shared by the three affected individuals.

### *LEMD2* c.T38G cosegregates with Hutterite‐type cataract in 84 of 84 individuals from four families

**Figure 5:**
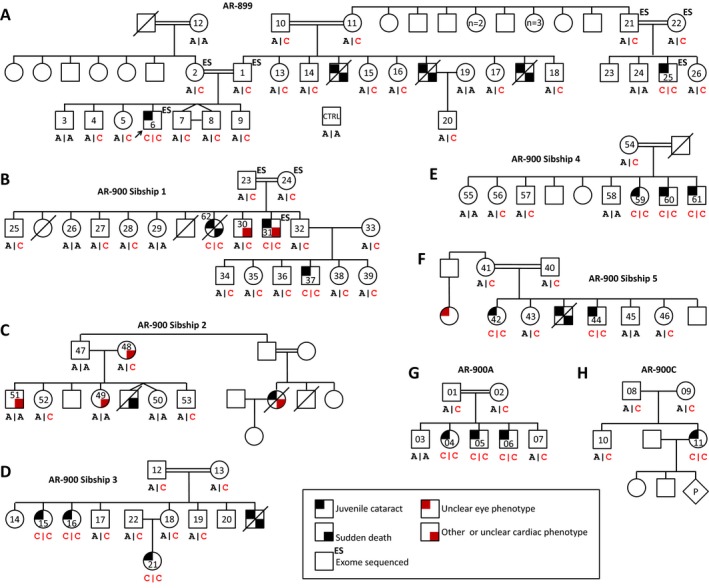
Hutterite‐type cataracts cosegregate with a homozygous *LEMD2* mutation (c.T38G) in 84 of 84 individuals from four families. (A–H) *LEMD2* c.T38G cosegregates perfectly with autosomal recessive cataracts in 84 subjects, including 17 affected individuals, from four Hutterite families (LOD = 9.62). Genotypes are in forward genomic orientation, shown below pedigree shapes (red text, mutant allele; black text, wt allele). Pedigrees have been trimmed to show only living generations/relevant individuals.

The *MUC21* and *LEMD2* variants both fall within the 9.5 Mb linked region on chromosome 6p (Fig. 3E), and both are homozygous, potentially damaging, and rare. Thus, additional studies were needed to determine which is the likely causal variant for Hutterite‐type cataract. *MUC21* c.665delC (Fig. S2) and *LEMD2* c.T38G (Fig. 4) were first genotyped in a single trio each, confirming their presence and proper zygosity. Sequencing a complete mutant amplicon of *MUC21* ensured that the reading frame is not restored upstream or downstream of this potential frameshift allele in exon 2 (Fig. S5). *LEMD2* c.T38G cosegregated perfectly with the cataract phenotype in a total of 84 individuals from four Hutterite families (Fig. 5); it is homozygous in 17 of 17 individuals with cataracts, heterozygous in 22 of 22 obligate carriers, and heterozygous or absent in 45 of 45 remaining family members (LOD = 9.62; one monozygotic twin pair is counted as a single birth/individual). Contrastingly, *MUC21* c.665delC does not cosegregate perfectly with cataracts (Fig. S6); one healthy individual (AR‐900‐57) is also homozygous for *MUC21* c.665delC. This individual was examined by portable biomicroscopy (R.A.L.) at age 47 years and his lenses are each normal for his age. The reading frame of *MUC21* is not restored upstream or downstream in exon 2 in this individual (data not shown).

### SNP‐based fine mapping further supports *LEMD2* – and excludes *MUC21* – as the Hutterite‐type cataract disease gene

**Figure 6:**
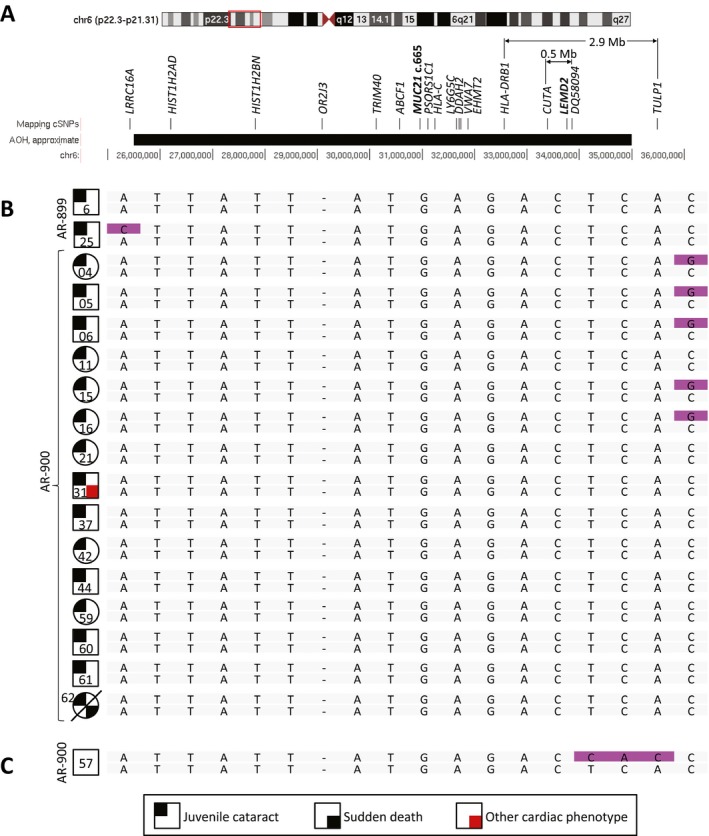
SNP‐based fine mapping refines the linked interval to a 0.5‐ to 2.9‐Mb region containing *LEMD2* but not *MUC21*. To map the Hutterite cataract locus more finely and to confirm an association with *LEMD2*, we performed SNP‐based fine mapping of the 9.5 Mb chromosome 6p region identified by autozygosity mapping. (A) Sixteen genotypable cSNPs span the region, named by the gene in which they reside (see Table S3 for dbSNP IDs). *MUC21* and *LEMD2* are also shown. (B) Genotyping of each SNP in all 17 affected individuals demonstrates homozygosity (gray) of the entire 9.5 Mb region, with boundaries determined by recombinations on either side (purple). Genotypes are shown for the mapping SNPs, *MUC21* c.665, and *LEMD2* c.38, spaced evenly and in genomic order. (C) Unaffected individual AR‐900‐57, while homozygous for the majority of SNPs in the region, is heterozygous for SNPs in *CUTA* and *DQ580984*, and *LEMD2* c.T38G. This defines a linked interval of 0.5–2.9 Mb (6p21.32‐p21.31; see A) and supports *LEMD2* as a candidate gene for Hutterite‐type cataract.

The above genotyping data support *LEMD2*, but not *MUC21*, as a candidate disease gene for Hutterite‐type cataract. To confirm this finding and to map the Hutterite‐type cataract locus more finely, we performed SNP‐based fine mapping of the 9.5 Mb region linked to cataracts. Sixteen SNPs spanning the region were selected based on WES data, such that each SNP would be informative (Fig. 6A; Table S3). Genotyping of each SNP in all 17 affected individuals demonstrated homozygosity of the entire 9.5 Mb linked region, with boundaries determined by recombinations on either side (Fig. 6B). Individual AR‐900‐57, while homozygous for the majority of SNPs in the region, possesses an informative recombination that renders him heterozygous for two SNPs toward its centromeric side (Fig. 6C). These SNPs thus define a subregion of 0.5–2.9 Mb (6p21.32‐p21.31; minimum interval chr6:33384473‐33851052, maximum interval chr6:32557483‐35479574) in which zygosity differs between AR‐900‐57 and the affected individuals. As *LEMD2*, but not *MUC21*, is within this narrower linked region, the data support *LEMD2* as a candidate gene for Hutterite‐type cataract, while excluding *MUC21*.

### *LEMD2* is a promising candidate gene

To appraise whether *LEMD2* exhibits the characteristics of a disease gene, we again queried the ExAC database. While a number of *LEMD2* variants exist among the 60,657 ExAC exomes, loss‐of‐function variants (LoF) are rare (Fig. S7), as may be expected for a candidate disease gene. Only five LoF variants were identified among the ExAC exomes (Table S4), all in the heterozygous state, and with a total allele frequency (*q*) of 0.000131 (*q* ^2^ = 1.71e‐8, or approximately 1 in 58 million). To confirm the rarity of *LEMD2* LoF mutations, we queried approximately 5700 exomes sequenced in the Baylor Miraca Genetics Laboratories (Yang et al. 2014) and approximately 4500 exomes sequenced as part of the BHCMG (Bamshad et al. 2012); no additional homozygous or compound heterozygous LoF variants were found in either database. We queried several in silico tools to predict the pathogenicity of the p.Leu13Arg variant in *LEMD2*. This variant is predicted to be damaging by SIFT (score = 0) (Ng and Henikoff 2003; Kumar et al. 2009), probably damaging by PolyPhen2 (score = 0.999) (Adzhubei et al. 2010), and disease causing by MutationTaster2 (score = 102) (Schwarz et al. 2014). The PHRED‐scaled score for the p.Leu13Arg *LEMD2* variant, computed with combined annotation‐dependent depletion (CADD) is 26.8, indicating that the predicted deleteriousness of this variant ranks between the top 0.1% and 1.0% of single nucleotide or indel variants within the human genome (Kircher et al. 2014).

To determine whether *LEMD2* is expressed in lens, and therefore potentially important for lens development or function, we queried two gene expression databases. In the iSYTE browser (Lachke et al. 2012), *LEMD2* is among the minority of genes in the 2.9 Mb linked region to be expressed in developing mouse lens (Fig. S8). This finding is replicated in data from newborn mouse lens (Hoang et al. 2014) (Table S5). By RT‐PCR, we identified consistent expression of *Lemd2* in mouse lenses in two strains (FVB/NJ and B57/6J) across various pre‐ and postnatal time points (Fig. S9). Additionally, RT‐PCR revealed expression of *LEMD2* in human whole lens and two lens compartments, as well as in the FHL124 lens epithelium cell line (Fig. S9).

### CNV analysis

Copy‐number variants (CNVs; i.e., large deletions or duplications) are not identified by standard exome sequencing analysis. To investigate the possibility that a causal CNV was overlooked, we utilized two complementary modalities to identify CNVs within the 9.5 Mb region linked to cataracts. First, a 1M probe genome‐wide comparative genomic hybridization (CGH) array was applied to DNA from one affected individual, AR‐899‐06. A single CNV was detected within the linked region: a low‐copy repeat (LCR)‐mediated duplication in homozygous form (Fig. S10). This CNV includes the centromeric half of *MICA*, the entirety of *HCP5* and *HCG26*, and the telomeric half of *MICB* (Fig. S10B). This CNV is common in human genome databases in the heterozygous state (MacDonald et al. 2014) and is outside of the narrow mapped interval at 6p21.32‐p21.31.

As the above array CGH assay has the power to detect only CNVs > ~10 kb in size, we also performed CNV analysis of exome data, a potentially higher resolution approach. Using an algorithm designed to detect homozygous and hemizygous deletions, we found that the three affected individuals shared no such deletion (Fig. S10E–F).

### Mutations in *DSC2* do not explain sudden cardiac death in the majority of sibships

Six of seven individuals experiencing sudden cardiac death had cataracts as children (AR‐899 and AR‐900 sibships 1, 3, and 5), while one (AR‐900 sibship 2) did not (Fig. 5). To explain this phenomenon, we considered the possibility that the deceased individual in AR‐900 sibship 2 possesses a distinct genetic form of sudden death.

*DSC2*, mapping to chromosome 18, is the gene for arrhythmogenic right ventricular dysplasia 11 (OMIM #610476). Gerull et al. described *DSC2* mutations (c.C1660T; p.Q554X) in Hutterite individuals with cardiac phenotypes (Gerull et al. 2013), including individuals in AR‐900 sibship 2 (Fig. S11). We genotyped this variant in parents of individuals who died suddenly, as well as one individual experiencing sudden death (AR‐900‐62). This variant is absent in AR‐900‐62 and in obligate carrier parents from three of five affected sibships (AR‐899 and AR‐900 sibships 1 and 5; Fig. S11). Only in AR‐900 sibship 2, as previously reported by Gerull et al. (2013), are parents each heterozygous and a homozygous individual with a cardiac phenotype is found. AR‐900 sibship 3 also contains a single carrier parent (AR‐900‐13), although the other parent (AR‐900‐12) is not a carrier of the pathogenic *DSC2* variant. These data indicate that the previously described *DSC2* variant may explain sudden cardiac death only in AR‐900 sibship 2. In contrast, the p.Leu13Arg *LEMD2* variant is present in a heterozygous state in all remaining obligate carrier parents (AR‐899 and AR‐900 sibships 1, 3, and 5; Fig. 5). This variant is also present in a homozygous state in AR‐900‐62, for whom juvenile cataracts and sudden cardiac death co‐occurred. Postmortem examination of individual AR‐900‐62 demonstrated myocardial scarring and fibrosis of the lateral left ventricle free wall in the setting of normally distributed coronary arteries and no detectable coronary thrombi or vascular lesions.

## Discussion

Hutterite‐type cataracts are autosomal recessive juvenile cataracts observed among the Hutterites of North America. Despite being identified nearly 30 years ago, the phenotype has been described in only a single pedigree and has not been investigated previously at either the genetic mapping or genomic sequencing level.

### Hutterite‐type cataracts map to a 9.5‐Mb region on chromosome 6p containing two candidate variants

We identified four Hutterite families with multiple individuals affected by juvenile‐onset cataracts. Exome sequencing of three trios allowed autozygosity mapping, which localized the phenotype to a 9.5‐Mb autozygous region on chromosome 6p22.2‐p21.31. This is the sole such region in the genome and contains no known isolated cataract genes nor mapped cataract loci, confirming that the Hutterite‐type cataract locus is a novel cataract locus. No potentially disease‐causing CNVs were identified in this region. Of note, a potential limitation of mapping by exome sequencing is that a nongenic or poorly sequenced region linked to a phenotype may not be discovered. However, the extremely high LOD score (9.62) of the *LEMD2* candidate variant (see below) suggests that this portion of 6p – to the exclusion of other regions of the genome – is in the sole region linked to cataracts.

Exome variant‐level data were subsequently mined, identifying two candidate variants: *MUC21* c.665delC, a predicted frameshift variant, and *LEMD2* c.T38G, a missense mutation of a conserved nucleotide in a key domain of LEMD2. As both variants are within the 9.5 Mb linked region on chromosome 6p, and both are homozygous, potentially damaging, and rare, additional studies were needed to determine which is the likely causal variant for Hutterite‐type cataract.

### The *LEMD2* mutation cosegregates perfectly with Hutterite‐type cataract

*MUC21* c.665delC and *LEMD2* c.T38G were genotyped in four large Hutterite‐type cataract families. *LEMD2* c.T38G cosegregated perfectly with the cataract phenotype in a total of 84 individuals including 17 of 17 individuals with cataracts (LOD = 9.62). This supports *LEMD2* as the Hutterite‐type cataract disease gene. Contrastingly, *MUC21* c.665delC does not cosegregate perfectly with cataracts, making it less likely that *MUC21* is the cataract gene.

### Fine mapping maps Hutterite‐type cataract to a <3 Mb subregion including *LEMD2*

SNP‐based fine mapping of chromosome 6p22.2‐p21.31 revealed that all 17 affected individuals were homozygous for the entire 9.5 Mb linked region. This region contains the major histocompatibility complex (MHC), which exhibits variable (Miretti et al. 2005) and, on average, increased (Cullen et al. 2002) linkage disequilibrium, leading to conserved, extended haplotypes in some individuals (Yunis et al. 2003; Vandiedonck and Knight 2009). This may explain the lack of informative recombinants among the 17 affected subjects. However, an informative recombination was found in AR‐900‐57, suggesting that the Hutterite‐type cataract gene resides within a 0.5‐ to 2.9‐Mb subregion containing *LEMD2* but not *MUC21* and strengthening the candidacy of *LEMD2* as the disease gene.

### Sudden death

Six individuals who had cataracts as children have died suddenly at an early age (22, 23, 26, 34, 37, and 42 years). This is a novel observation. Some members of AR‐900 have been found to have a mutation in *DSC2* (c.C1660T) (Gerull et al. 2013), and we find that Sanger sequencing for this variant in obligate carrier parents of the sudden cardiac death phenotype confirms the presence of the *DSC2* c.C1660T variant in AR‐900 sibship 2, for which sudden cardiac death in the absence of juvenile cataracts has been reported. However, we excluded the presence of homozygous *DSC2* c.C1660T in all sibships for which sudden death and cataracts co‐occur, confirming that an alternative genetic etiology (e.g., juvenile cataracts and sudden death are both caused by mutations in *LEMD2*) exists. Although there are 16 individuals predicted to be at risk for a sudden cardiac event based on their history of juvenile cataracts, variable age of onset and reduced penetrance are well‐known features of inherited cardiomyopathies (Teekakirikul et al. 2013), and only three of these individuals have surpassed the age of the oldest individual (AR‐900‐62) reported to have both cataracts and sudden cardiac death.

### LEMD2

LEM (LAP2, emerin, MAN1) domain‐containing proteins localize to the inner nuclear membrane, interact with the nuclear lamina, and are involved in nuclear membrane organization (Lin et al. 2000; Cai et al. 2001; Laguri et al. 2001). The LEM domain is conserved across species including *D. melanogaster* and *C. elegans*, and shared among the human proteins LEMD2, emerin, MAN1 (encoded by *LEMD3*), LAP2 (encoded by *TMPO*), ANKLE1, and ANKLE2 (Harris et al. 1994; Furukawa et al. 1995; Berger et al. 1996; Dechat et al. 1998; Lin et al. 2000). LEMD2, or LEM domain‐containing protein 2, is a ubiquitously expressed 503‐amino acid protein encoded by *LEMD2* (previously *NET25*) and consisting of an N‐terminal LEM domain, a C‐terminal MSC (MAN1‐Src1p C‐terminal) domain, and two transmembrane domains (Brachner et al. 2005; Huber et al. 2009). LEMD2 itself has been demonstrated to localize to the nuclear envelope, and its N‐terminal and transmembrane domains are required for this localization (Brachner et al. 2005). We now describe an association between a homozygous *LEMD2* missense (p.Leu13Arg) variant affecting a highly conserved residue of the LEM domain and a phenotype of Hutterite‐type juvenile cataracts associated with sudden cardiac death.

*LEMD2* has been proposed as a novel disease gene candidate based on the role of several nuclear envelope‐associated genes in human diseases, particularly laminopathies and muscular dystrophies (Brachner et al. 2005; Huber et al. 2009). Of particular relevance is the relationship between LEMD2, emerin, and A‐type lamins. The nuclear envelope itself includes the nuclear lamina composed of lamin intermediate filaments, the inner and outer membranes, and the nuclear pore complexes. The dysfunction of the nuclear envelope can lead to a variety of phenotypes, including abnormalities in muscle function and cardiac arrhythmia (Worman and Bonne 2007; Mendez‐Lopez and Worman 2012). Both LEMD2 and emerin have been shown to interact directly with A‐type lamins, which are necessary for their proper localization to the inner nuclear membrane, providing a basis for overlapping phenotypes among these proteins (Sullivan et al. 1999; Clements et al. 2000; Vaughan et al. 2001; Holaska et al. 2003; Brachner et al. 2005). All three proteins are required for myogenesis, and overexpression of LEMD2 can rescue impaired myogenic differentiation caused by emerin expression knockdown in vitro, suggesting overlapping functions – and perhaps overlapping phenotypes – for these two proteins (Frock et al. 2006; Huber et al. 2009). Pathogenic variants in *EMD*, the gene encoding emerin, cause X‐linked Emery–Dreifuss muscular dystrophy (EDMD) affecting both skeletal and cardiac muscle, resulting in cardiac conduction defects that may lead to sudden cardiac death (Bione et al. 1994). A more recent study of patients with EDMD or similar phenotypes demonstrated an association between muscular dystrophy and pathogenic variants in *SUN1* and *SUN2*, which encode SUN proteins that localize to the inner nuclear membrane and form part of the LINC (Linker of Nucleoskeleton and Cytoskeleton) complex (Padmakumar et al. 2005; Crisp et al. 2006; Haque et al. 2006; Meinke et al. 2014). Human variation in *LMNA*, the gene encoding lamin A and lamin C, has been associated with numerous phenotypes, including Emery–Dreifuss and other forms of muscular dystrophy, dilated cardiomyopathy, neuropathy, Hutchinson–Gilford progeria, and lipodystrophy (Worman and Bonne 2007). These findings provide strong support for the role of LEMD2 in cardiac development, cardiomyopathy, and sudden cardiac death. Lemd2 is required for mouse development, and mice homozygous for a disrupted *Lemd2* allele are embryonic lethal by E11.5 (Tapia et al. 2015). Studies of these embryos at E10.5 demonstrated a thin myocardium with underdeveloped trabeculae, consistent with a role for LEMD2 in cardiac development (Tapia et al. 2015).

The downstream effects of loss of LEMD2 provide some evidence for a connection between LEMD2 and cataract development. Downregulation of mouse Lemd2 by RNAi in myoblast cultures resulted in increased phosphorylation of MAP kinases Erk1/2 and Jnk, and this regulatory interaction was dependent on the N terminus of Lemd2, which includes the LEM domain (Tapia et al. 2015). The MAP kinase pathway is known to play a role in cataract development, and transgenic mice expressing a constitutively active form of MEK1, an upstream activator of Erk1/2, develop cataracts and macrophthalmia (Gong et al. 2001). More recent functional studies demonstrated that ERK activation is required for lens fiber differentiation (Le and Musil 2001; Lovicu and McAvoy 2001; Golestaneh et al. 2004). These findings are consistent with a role for LEMD2, and in particular the LEM domain of LEMD2, in cardiomyopathy and cataract development. Lens gene expression databases and RT‐PCR experiments in the present study confirm the expression of *Lemd2* and *LEMD2* in mouse and human lenses, respectively.

Our report of a pathogenic *LEMD2* variant involving a highly conserved leucine residue that is conserved across human LEM domain‐containing proteins (Fig. 4E–F) (Brachner et al. 2005) provides additional support for the candidacy of *LEMD2* as a novel disease gene. The c.T38G mutation is predicted to be damaging, mutating a conserved nucleotide in the LEM domain of *LEMD2*. Commensurate with the rarity of nonsyndromic juvenile cataracts (Wirth et al. 2002), loss‐of‐function (LoF) variants in *LEMD2* are extremely uncommon, and homozygous or compound heterozygous LoF *LEMD2* variants have not been reported outside of the extended Hutterite family currently under study.

### Mucin 21

Mucin genes code for cell surface or secreted proteins important for epithelial function and overexpressed in some carcinomas (e.g., *MUC16*, which codes for CA‐125). Mucin proteins contain a single‐pass transmembrane domain, a long mucin domain projecting outside of the cell, and a signal peptide (Fig. S2D). The mucin domain consists of a variable number of tandem repeats (VNTR), each repeat being of fixed length within a given mucin (to maintain reading frame) but often of variable sequence. The mucin domain becomes heavily O‐glycosylated, allowing for the hydration/gel formation exemplified by mucus, of which mucins are a part and from which they derive their name. While our data rule out *MUC21* from being the gene for Hutterite‐type cataract, it is interesting that no obvious clinical phenotype results from the homozygous c.665delC variant, a predicted frameshift allele in the mucin domain VNTR of *MUC21*.

*MUC21* was identified as the likely ortholog of the gene coding for epiglycanin (Itoh et al. 2008), a murine cell‐surface mucin identified in mouse mammary carcinoma TA3‐Ha cells (Codington et al. 1972, 1975). TA3‐Ha cells are capable of allogeneic growth (Friberg 1972), proposed to result from epiglycanin preventing detection by immune cells (summarized by Itoh et al. 2008). This intriguingly suggests the potential of tumor protective properties of human *MUC21* knockouts (e.g., the homozygous c.665delC subjects presented here). Whether this or any other unnoticed phenotype exists in these individuals is a possible line of future investigation.

### Summary

We performed a genetic study of Hutterite‐type cataract. Whole exome sequencing data enabled genome‐wide autozygosity mapping, which localized the disease locus to a 9.5 Mb region of chromosome 6p containing two candidate variants (*LEMD2* c.T38G and *MUC21* c.665delC). While the *LEMD2* variant cosegregated perfectly with cataracts, the *MUC21* variant did not. SNP‐based fine mapping within the 9.5 Mb linked region confirmed this finding by refining the locus to a 0.5‐ to 2.9‐Mb subregion (6p21.32‐p21.31) containing *LEMD2* but not *MUC21*. The mutation in *LEMD2* is predicted to disrupt a conserved position of a key domain, and *LEMD2* is expressed in the lens. These data suggest that *LEMD2* is the disease gene for Hutterite‐type cataract. Finally, we observed that the most common cause of death in individuals with cataracts is sudden cardiac death at an early age, suggesting an association of this phenotype with Hutterite‐type cataracts.

## Conflict of Interest

The Department of Molecular and Human Genetics at Baylor College of Medicine derives revenue from molecular genetic testing offered in the Baylor Miraca Genetics Laboratories.

## Supporting information


**Figure S1.** A single autozygous region segregates with Hutterite‐type cataracts, confirming linkage to chromosome 6p22.2‐p21.31.
**Figure S2.** *MUC21* c.665delC is confirmed by Sanger sequencing and represents a predicted frameshift allele.
**Figure S3.** Predicted translational effect of *MUC21* c.665delC.
**Figure S4.** The vast majority of exons within the 9.5 Mb linked region are covered ≥20×.
**Figure S5.** Complete sequences of *MUC21* exon 2.
**Figure S6.** Hutterite‐type cataracts cosegregate imperfectly with a homozygous *MUC21* variant (c.665delC).
**Figure S7.** *LEMD2* harbors few loss‐of‐function alleles.
**Figure S8.** *LEMD2* is among the minority of genes within chromosome 6p21.32‐p21.31 expressed in the developing mouse lens.
**Figure S9.** Expression of *LEMD2* and its murine ortholog, *Lemd2*, in the lens.
**Figure S10.** CNV analysis within the 9.5 Mb region linked to Hutterite cataract reveals no potential causative CNVs.
**Figure S11.** *DSC2* c.C1660T does not segregate with sudden cardiac death in the majority of cataract sibships with sudden death.
**Table S1.** Characteristics of Hutterite‐type cataracts in all reported individuals.
**Table S2.** Primer sequences.
**Table S3.** cSNPs used to fine‐map the cataract locus within 6p22.2‐p21.31.
**Table S4.** Loss‐of‐function Alleles in *LEMD2* in the Exome Aggregation Consortium (ExAC) Browser.
**Table S5.** Genes within the 2.9 Mb linked region that are expressed in mouse lens (data from Hoang et al. 2014).

## References

[mgg3181-bib-0001] Adzhubei, I. A. , S. Schmidt , L. Peshkin , V. E. Ramensky , A. Gerasimova , P. Bork , et al. 2010 A method and server for predicting damaging missense mutations. Nat. Methods 7:248–249. 20354512 10.1038/nmeth0410-248 PMC2855889

[mgg3181-bib-0002] Bainbridge, M. N. , M. Wang , D. L. Burgess , C. Kovar , M. J. Rodesch , M. D'Ascenzo , et al. 2010 Whole exome capture in solution with 3 Gbp of data. Genome Biol. 11:R62. 20565776 10.1186/gb-2010-11-6-r62 PMC2911110

[mgg3181-bib-0003] Bainbridge, M. N. , M. Wang , Y. Wu , I. Newsham , D. M. Muzny , J. L. Jefferies , et al. 2011 Targeted enrichment beyond the consensus coding DNA sequence exome reveals exons with higher variant densities. Genome Biol. 12:R68. 21787409 10.1186/gb-2011-12-7-r68 PMC3218830

[mgg3181-bib-0004] Bamshad, M. J. , J. A. Shendure , D. Valle , A. Hamosh , J. R. Lupski , R. A. Gibbs , et al. 2012 The Centers for Mendelian Genomics: a new large‐scale initiative to identify the genes underlying rare Mendelian conditions. Am. J. Med. Genet. A 158A:1523–1525. 22628075 10.1002/ajmg.a.35470 PMC3702263

[mgg3181-bib-0005] Berger, R. , L. Theodor , J. Shoham , E. Gokkel , F. Brok‐Simoni , K. B. Avraham , et al. 1996 The characterization and localization of the mouse thymopoietin/lamina‐associated polypeptide 2 gene and its alternatively spliced products. Genome Res. 6:361–370. 8743987 10.1101/gr.6.5.361

[mgg3181-bib-0006] Bione, S. , E. Maestrini , S. Rivella , M. Mancini , S. Regis , G. Romeo , et al. 1994 Identification of a novel X‐linked gene responsible for Emery‐Dreifuss muscular dystrophy. Nat. Genet. 8:323–327. 7894480 10.1038/ng1294-323

[mgg3181-bib-0007] Brachner, A. , S. Reipert , R. Foisner , and J. Gotzmann . 2005 LEM2 is a novel MAN1‐related inner nuclear membrane protein associated with A‐type lamins. J. Cell Sci. 118:5797–5810. 16339967 10.1242/jcs.02701

[mgg3181-bib-0008] Cai, M. , Y. Huang , R. Ghirlando , K. L. Wilson , R. Craigie , and G. M. Clore . 2001 Solution structure of the constant region of nuclear envelope protein LAP2 reveals two LEM‐domain structures: one binds BAF and the other binds DNA. EMBO J. 20:4399–4407. 11500367 10.1093/emboj/20.16.4399 PMC125263

[mgg3181-bib-0009] Carr, I. M. , S. Bhaskar , J. O'Sullivan , M. A. Aldahmesh , H. E. Shamseldin , A. F. Markham , et al. 2013 Autozygosity mapping with exome sequence data. Hum. Mutat. 34:50–56. 23090942 10.1002/humu.22220

[mgg3181-bib-0010] Challis, D. , J. Yu , U. S. Evani , A. R. Jackson , S. Paithankar , C. Coarfa , et al. 2012 An integrative variant analysis suite for whole exome next‐generation sequencing data. BMC Bioinformatics 13:8. 22239737 10.1186/1471-2105-13-8 PMC3292476

[mgg3181-bib-0011] Clements, L. , S. Manilal , D. R. Love , and G. E. Morris . 2000 Direct interaction between emerin and lamin A. Biochem. Biophys. Res. Commun. 267:709–714. 10673356 10.1006/bbrc.1999.2023

[mgg3181-bib-0012] Codington, J. F. , B. H. Sanford , and R. W. Jeanloz . 1972 Glycoprotein coat of the TA3 cell. Isolation and partial characterization of a sialic acid containing glycoprotein fraction. Biochemistry 11:2559–2564. 5065218 10.1021/bi00764a001

[mgg3181-bib-0013] Codington, J. F. , K. B. Linsley , R. W. Jeanloz , T. Irimura , and T. Osawa . 1975 Immunochemical and chemical investigations of the structure of glycoprotein fragments obtained from epiglycanin, a glycoprotein at the surface of the TA3‐Ha cancer cell. Carbohydr. Res. 40:171–182. 1125949 10.1016/s0008-6215(00)82679-8

[mgg3181-bib-0014] Cottingham, R. W. Jr , R. M. Idury , and A. A. Schäffer . 1993 Faster sequential genetic linkage computations. Am. J. Hum. Genet. 53:252–263. 8317490 PMC1682239

[mgg3181-bib-0015] Crisp, M. , Q. Liu , K. Roux , J. B. Rattner , C. Shanahan , B. Burke , et al. 2006 Coupling of the nucleus and cytoplasm: role of the LINC complex. J. Cell Biol. 172:41–53. 16380439 10.1083/jcb.200509124 PMC2063530

[mgg3181-bib-0016] Cullen, M. , S. P. Perfetto , W. Klitz , G. Nelson , and M. Carrington . 2002 High‐resolution patterns of meiotic recombination across the human major histocompatibility complex. Am. J. Hum. Genet. 71:759–776. 12297984 10.1086/342973 PMC378534

[mgg3181-bib-0017] Dechat, T. , J. Gotzmann , A. Stockinger , C. A. Harris , M. A. Talle , J. J. Siekierka , et al. 1998 Detergent‐salt resistance of LAP2alpha in interphase nuclei and phosphorylation‐dependent association with chromosomes early in nuclear assembly implies functions in nuclear structure dynamics. EMBO J. 17:4887–4902. 9707448 10.1093/emboj/17.16.4887 PMC1170818

[mgg3181-bib-0018] Exome Aggregation Consortium (ExAC) , Cambridge, MA Available at http://exac.broadinstitute.org (accessed 19 July 2015).

[mgg3181-bib-0019] Francis, P. J. , and A. T. Moore . 2004 Genetics of childhood cataract. Curr. Opin. Ophthalmol. 15:10–15. 14743013 10.1097/00055735-200402000-00003

[mgg3181-bib-0020] Francis, P. J. , V. Berry , S. S. Bhattacharya , and A. T. Moore . 2000 The genetics of childhood cataract. J. Med. Genet. 37:481–488. 10882749 10.1136/jmg.37.7.481 PMC1734631

[mgg3181-bib-0021] Friberg, S. Jr . 1972 Comparison of an immunoresistant and an immunosusceptible ascites subline from murine tumor TA3. I. Transplantability, morphology, and some physicochemical characteristics. J. Natl Cancer Inst. 48:1463–1476. 5030958

[mgg3181-bib-0022] Frock, R. L. , B. A. Kudlow , A. M. Evans , S. A. Jameson , S. D. Hauschka , and B. K. Kennedy . 2006 Lamin A/C and emerin are critical for skeletal muscle satellite cell differentiation. Genes Dev. 20:486–500. 16481476 10.1101/gad.1364906 PMC1369050

[mgg3181-bib-0023] Furukawa, K. , N. Pante , U. Aebi , and L. Gerace . 1995 Cloning of a cDNA for lamina‐associated polypeptide 2 (LAP2) and identification of regions that specify targeting to the nuclear envelope. EMBO J. 14:1626–1636. 7737115 10.1002/j.1460-2075.1995.tb07151.x PMC398255

[mgg3181-bib-0024] Gerull, B. , F. Kirchner , J. X. Chong , J. Tagoe , K. Chandrasekharan , O. Strohm , et al. 2013 Homozygous founder mutation in desmocollin‐2 (*DSC2*) causes arrhythmogenic cardiomyopathy in the Hutterite population. Circ. Cardiovasc. Genet. 6:327–336. 23863954 10.1161/CIRCGENETICS.113.000097

[mgg3181-bib-0025] Golestaneh, N. , J. Fan , R. N. Fariss , W. K. Lo , P. S. Zelenka , and A. B. Chepelinsky . 2004 Lens major intrinsic protein (MIP)/aquaporin 0 expression in rat lens epithelia explants requires fibroblast growth factor‐induced ERK and JNK signaling. J. Biol. Chem. 279:31813–31822. 15145928 10.1074/jbc.M403473200

[mgg3181-bib-0026] Gong, X. , X. Wang , J. Han , I. Niesman , Q. Huang , and J. Horwitz . 2001 Development of cataractous macrophthalmia in mice expressing an active MEK1 in the lens. Invest. Ophthalmol. Vis. Sci. 42:539–548. 11222509

[mgg3181-bib-0027] Gonzaga‐Jauregui, C. , F. Zhang , C. F. Towne , S. D. Batish , and J. R. Lupski . 2010 *GJB1*/Connexin 32 whole gene deletions in patients with X‐linked Charcot‐Marie‐Tooth disease. Neurogenetics 11:465–470. 20532933 10.1007/s10048-010-0247-4 PMC4222676

[mgg3181-bib-0028] Haque, F. , D. J. Lloyd , D. T. Smallwood , C. L. Dent , C. M. Shanahan , A. M. Fry , et al. 2006 SUN1 interacts with nuclear lamin A and cytoplasmic nesprins to provide a physical connection between the nuclear lamina and the cytoskeleton. Mol. Cell. Biol. 26:3738–3751. 16648470 10.1128/MCB.26.10.3738-3751.2006 PMC1488999

[mgg3181-bib-0029] Harris, C. A. , P. J. Andryuk , S. Cline , H. K. Chan , A. Natarajan , J. J. Siekierka , et al. 1994 Three distinct human thymopoietins are derived from alternatively spliced mRNAs. Proc. Natl Acad. Sci. USA 91:6283–6287. 7517549 10.1073/pnas.91.14.6283 PMC44185

[mgg3181-bib-0030] Hoang, T. V. , P. K. R. Kumar , S. Sutharzan , P. A. Tsonis , C. Liang , and M. L. Robinson . 2014 Comparative transcriptome analysis of epithelial and fiber cells in newborn mouse lenses with RNA sequencing. Mol. Vis. 20:1491–1517. 25489224 PMC4225139

[mgg3181-bib-0031] Holaska, J. M. , K. K. Lee , A. K. Kowalski , and K. L. Wilson . 2003 Transcriptional repressor germ cell‐less (GCL) and barrier to autointegration factor (BAF) compete for binding to emerin in vitro. J. Biol. Chem. 278:6969–6975. 12493765 10.1074/jbc.M208811200

[mgg3181-bib-0501] Hostetler, J. A. , and G. E. Huntington . 1980 The Hutterites in North America. Holt, Rinehart, and Winston. New York.

[mgg3181-bib-0032] Huber, M. D. , T. Guan , and L. Gerace . 2009 Overlapping functions of nuclear envelope proteins NET25 (Lem2) and emerin in regulation of extracellular signal‐regulated kinase signaling in myoblast differentiation. Mol. Cell. Biol. 29:5718–5728. 19720741 10.1128/MCB.00270-09 PMC2772735

[mgg3181-bib-0033] Itoh, Y. , M. Kamata‐Sakurai , K. Denda‐Nagai , S. Nagai , M. Tsuiji , K. Ishii‐Schrade , et al. 2008 Identification and expression of human epiglycanin/MUC21: a novel transmembrane mucin. Glycobiology 18:74–83. 17977904 10.1093/glycob/cwm118

[mgg3181-bib-0034] Kircher, M. , D. M. Witten , P. Jain , B. J. O'Roak , G. M. Cooper , and J. Shendure . 2014 A general framework for estimating the relative pathogenicity of human genetic variants. Nat. Genet. 46:310–315. 24487276 10.1038/ng.2892 PMC3992975

[mgg3181-bib-0035] Kumar, P. , S. Henikoff , and P. C. Ng . 2009 Predicting the effects of coding non‐synonymous variants on protein function using the SIFT algorithm. Nat. Protoc. 4:1073–1081. 19561590 10.1038/nprot.2009.86

[mgg3181-bib-0036] Lachke, S. A. , J. W. Ho , G. V. Kryukov , D. J. O'Connell , A. Aboukhalil , M. L. Bulyk , et al. 2012 iSyTE: integrated Systems Tool for Eye gene discovery. Invest. Ophthalmol. Vis. Sci. 53:1617–1627. 22323457 10.1167/iovs.11-8839 PMC3339920

[mgg3181-bib-0037] Laguri, C. , B. Gilquin , N. Wolff , R. Romi‐Lebrun , K. Courchay , I. Callebaut , et al. 2001 Structural characterization of the LEM motif common to three human inner nuclear membrane proteins. Structure 9:503–511. 11435115 10.1016/s0969-2126(01)00611-6

[mgg3181-bib-0038] Le, A. C. , and L. S. Musil . 2001 A novel role for FGF and extracellular signal‐regulated kinase in gap junction‐mediated intercellular communication in the lens. J. Cell Biol. 154:197–216. 11449001 10.1083/jcb.200101057 PMC2196873

[mgg3181-bib-0039] Lin, F. , D. L. Blake , I. Callebaut , I. S. Skerjanc , L. Holmer , M. W. McBurney , et al. 2000 MAN1, an inner nuclear membrane protein that shares the LEM domain with lamina‐associated polypeptide 2 and emerin. J. Biol. Chem. 275:4840–4847. 10671519 10.1074/jbc.275.7.4840

[mgg3181-bib-0040] Lovicu, F. J. , and J. W. McAvoy . 2001 FGF‐induced lens cell proliferation and differentiation is dependent on MAPK (ERK1/2) signalling. Development 128:5075–5084. 11748143 10.1242/dev.128.24.5075

[mgg3181-bib-0041] Lupski, J. R. , C. Gonzaga‐Jauregui , Y. Yang , M. N. Bainbridge , S. Jhangiani , C. J. Buhay , et al. 2013 Exome sequencing resolves apparent incidental findings and reveals further complexity of SH3TC2 variant alleles causing Charcot‐Marie‐Tooth neuropathy. Genome Med. 5:57. 23806086 10.1186/gm461 PMC3706849

[mgg3181-bib-0042] MacDonald, J. R. , R. Ziman , R. K. Yuen , L. Feuk , and S. W. Scherer . 2014 The Database of Genomic Variants: a curated collection of structural variation in the human genome. Nucleic Acids Res. 42:D986–D992. 24174537 10.1093/nar/gkt958 PMC3965079

[mgg3181-bib-0043] Meinke, P. , E. Mattioli , F. Haque , S. Antoku , M. Columbaro , K. R. Straatman , et al. 2014 Muscular dystrophy‐associated SUN1 and SUN2 variants disrupt nuclear‐cytoskeletal connections and myonuclear organization. PLoS Genet. 10:e1004605. 25210889 10.1371/journal.pgen.1004605 PMC4161305

[mgg3181-bib-0044] Mendez‐Lopez, I. , and H. J. Worman . 2012 Inner nuclear membrane proteins: impact on human disease. Chromosoma 121:153–167. 22307332 10.1007/s00412-012-0360-2

[mgg3181-bib-0045] Miretti, M. M. , E. C. Walsh , X. Ke , M. Delgado , M. Griffiths , S. Hunt , et al. 2005 A high‐resolution linkage‐disequilibrium map of the human major histocompatibility complex and first generation of tag single‐nucleotide polymorphisms. Am. J. Hum. Genet. 76:634–646. 15747258 10.1086/429393 PMC1199300

[mgg3181-bib-0046] Ng, P. C. , and S. Henikoff . 2003 SIFT: predicting amino acid changes that affect protein function. Nucleic Acids Res. 31:3812–3814. 12824425 10.1093/nar/gkg509 PMC168916

[mgg3181-bib-0047] Padmakumar, V. C. , T. Libotte , W. Lu , H. Zaim , S. Abraham , A. A. Noegel , et al. 2005 The inner nuclear membrane protein Sun1 mediates the anchorage of Nesprin‐2 to the nuclear envelope. J. Cell Sci. 118:3419–3430. 16079285 10.1242/jcs.02471

[mgg3181-bib-0048] Pearce, W. G. , J. A. Mackay , T. M. Holmes , K. Morgan , S. B. Fowlow , M. H. K. Shokeir , et al. 1987 Autosomal recessive juvenile cataract in Hutterites. Ophthalmic. Paediatr. Genet. 8:119–124. 3658338 10.3109/13816818709028527

[mgg3181-bib-0049] Reid, J. G. , A. Carroll , N. Veeraraghavan , M. Dahdouli , A. Sundquist , A. English , et al. 2014 Launching genomics into the cloud: deployment of *Mercury*, a next generation sequence analysis pipeline. BMC Bioinformatics 15:30. 24475911 10.1186/1471-2105-15-30 PMC3922167

[mgg3181-bib-0050] Schwarz, J. M. , D. N. Cooper , M. Schuelke , and D. Seelow . 2014 MutationTaster2: mutation prediction for the deep‐sequencing age. Nat. Methods 11:361–362. 24681721 10.1038/nmeth.2890

[mgg3181-bib-0051] Shiels, A. , and J. F. Hejtmancik . 2013 Genetics of human cataract. Clin. Genet. 84:120–127. 23647473 10.1111/cge.12182 PMC3991604

[mgg3181-bib-0052] Shiels, A. , T. M. Bennett , and J. F. Hejtmancik . 2010 Cat‐Map: putting cataract on the map. Mol. Vis. 16:2007–2015. 21042563 PMC2965572

[mgg3181-bib-0053] Shokeir, M. H. K. , and R. B. Lowry . 1985 Juvenile cataract in Hutterites. Am. J. Med. Genet. 22:495–500. 4061486 10.1002/ajmg.1320220307

[mgg3181-bib-0054] Sullivan, T. , D. Escalante‐Alcalde , H. Bhatt , M. Anver , N. Bhat , K. Nagashima , et al. 1999 Loss of A‐type lamin expression compromises nuclear envelope integrity leading to muscular dystrophy. J. Cell Biol. 147:913–920. 10579712 10.1083/jcb.147.5.913 PMC2169344

[mgg3181-bib-0055] Tapia, O. , L. G. Fong , M. D. Huber , S. G. Young , and L. Gerace . 2015 Nuclear envelope protein Lem2 is required for mouse development and regulates MAP and AKT kinases. PLoS ONE 10:e0116196. 25790465 10.1371/journal.pone.0116196 PMC4366207

[mgg3181-bib-0056] Teekakirikul, P. , M. A. Kelly , H. L. Rehm , N. K. Lakdawala , and B. H. Funke . 2013 Inherited cardiomyopathies: molecular genetics and clinical genetic testing in the postgenomic era. J. Mol. Diagn. 15:158–170. 23274168 10.1016/j.jmoldx.2012.09.002

[mgg3181-bib-0057] UCSC Genome Browser . Santa Cruz, CA Available at http://genome.ucsc.edu/ (accessed 19 July 2015).

[mgg3181-bib-0058] Vandiedonck, C. , and J. C. Knight . 2009 The human Major Histocompatibility Complex as a paradigm in genomics research. Brief Funct. Genomic. Proteomic. 8:379–394. 19468039 10.1093/bfgp/elp010 PMC2987720

[mgg3181-bib-0059] Vaughan, A. , M. Alvarez‐Reyes , J. M. Bridger , J. L. Broers , F. C. Ramaekers , M. Wehnert , et al. 2001 Both emerin and lamin C depend on lamin A for localization at the nuclear envelope. J. Cell Sci. 114:2577–2590. 11683386 10.1242/jcs.114.14.2577

[mgg3181-bib-0060] Wirth, M. G. , I. M. Russell‐Eggitt , J. E. Craig , J. E. Elder , and D. A. Mackey . 2002 Aetiology of congenital and paediatric cataract in an Australian population. Br. J. Ophthalmol. 86:782–786. 12084750 10.1136/bjo.86.7.782 PMC1771196

[mgg3181-bib-0061] Worman, H. J. , and G. Bonne . 2007 “Laminopathies”: a wide spectrum of human diseases. Exp. Cell Res. 313:2121–2133. 17467691 10.1016/j.yexcr.2007.03.028 PMC2964355

[mgg3181-bib-0062] Yang, Y. , D. M. Muzny , F. Xia , Z. Niu , R. Person , Y. Ding , et al. 2014 Molecular findings among patients referred for clinical whole‐exome sequencing. JAMA 312:1870–1879. 25326635 10.1001/jama.2014.14601 PMC4326249

[mgg3181-bib-0063] Yunis, E. J. , C. E. Larsen , M. Fernandez‐Vina , Z. L. Awdeh , T. Romero , J. A. Hansen , et al. 2003 Inheritable variable sizes of DNA stretches in the human MHC: conserved extended haplotypes and their fragments or blocks. Tissue Antigens 62:1–20. 12859592 10.1034/j.1399-0039.2003.00098.x

