# Supplementary material for: Hutterite‐type cataract maps to chromosome 6p21.32‐p21.31, cosegregates with a homozygous mutation in LEMD2, and is associated with sudden cardiac death
Source: Mol Genet Genomic Med. 2015 Nov 14;4(1):77–94. doi: 10.1002/mgg3.181 (PMC4707028; doi:10.1002/mgg3.181)
Supplement: Supplementary file 1 — Figure S1. A single autozygous region segregates with Hutterite‐type cataracts, confirming linkage to chromosome 6p22.2‐p21.31. Figure S2. MUC21 c.665delC is confirmed by Sanger sequencing and represents a predicted frameshift allele. Figure S3. Predicted translational effect of MUC21 c.665delC. Figure S4. The vast majority of exons within the 9.5 Mb linked region are covered ≥20×. Figure S5. Complete sequences of MUC21 exon 2. Figure S6. Hutterite‐type cataracts cosegregate imperfectly with a homozygous MUC21 variant (c.665delC). Figure S7. LEMD2 harbors few loss‐of‐function alleles. Figure S8. LEMD2 is among the minority of genes within chromosome 6p21.32‐p21.31 expressed in the developing mouse lens. Figure S9. Expression of LEMD2 and its murine ortholog, Lemd2, in the lens. Figure S10. CNV analysis within the 9.5 Mb region linked to Hutterite cataract reveals no potential causative CNVs. Figure S11. DSC2 c.C1660T does not segregate with sudden cardiac death in the majority of cataract sibships with sudden death. Table S1. Characteristics of Hutterite‐type cataracts in all reported individuals. Table S2. Primer sequences. Table S3. cSNPs used to fine‐map the cataract locus within 6p22.2‐p21.31. Table S4. Loss‐of‐function Alleles in LEMD2 in the Exome Aggregation Consortium (ExAC) Browser. Table S5. Genes within the 2.9 Mb linked region that are expressed in mouse lens (data from Hoang et al. 2014). [file MGG3-4-077-s001.pdf]

Supplement

Hutterite-type cataract maps to chromosome 6p21.32-p21.31, co-segregates with a homozygous mutation in *LEMD2*, and is associated with sudden cardiac death

Philip M. Boone, M.D., Ph.D., Bo Yuan, Ph.D., Shen Gu, Ph.D., Zhiwei Ma, M.D., Ph.D., Tomasz Gambin, Ph.D., Claudia Gonzaga-Jauregui, Ph.D., Mahim Jain, M.D., Ph.D., Todd J. Murdock, M.D., Janson J. White, B.S. Shalini N. Jhangiani, M.S., Kimberly Walker, M.S., Qiaoyan Wang, M.A., Donna M. Muzny, M.S., Richard A. Gibbs, Ph.D., J. Fielding Hejtmancik, M.D., Ph.D., James R. Lupski, M.D., Ph.D., Jennifer E. Posey, M.D., Ph.D., Richard Alan Lewis, M.D., M.S.

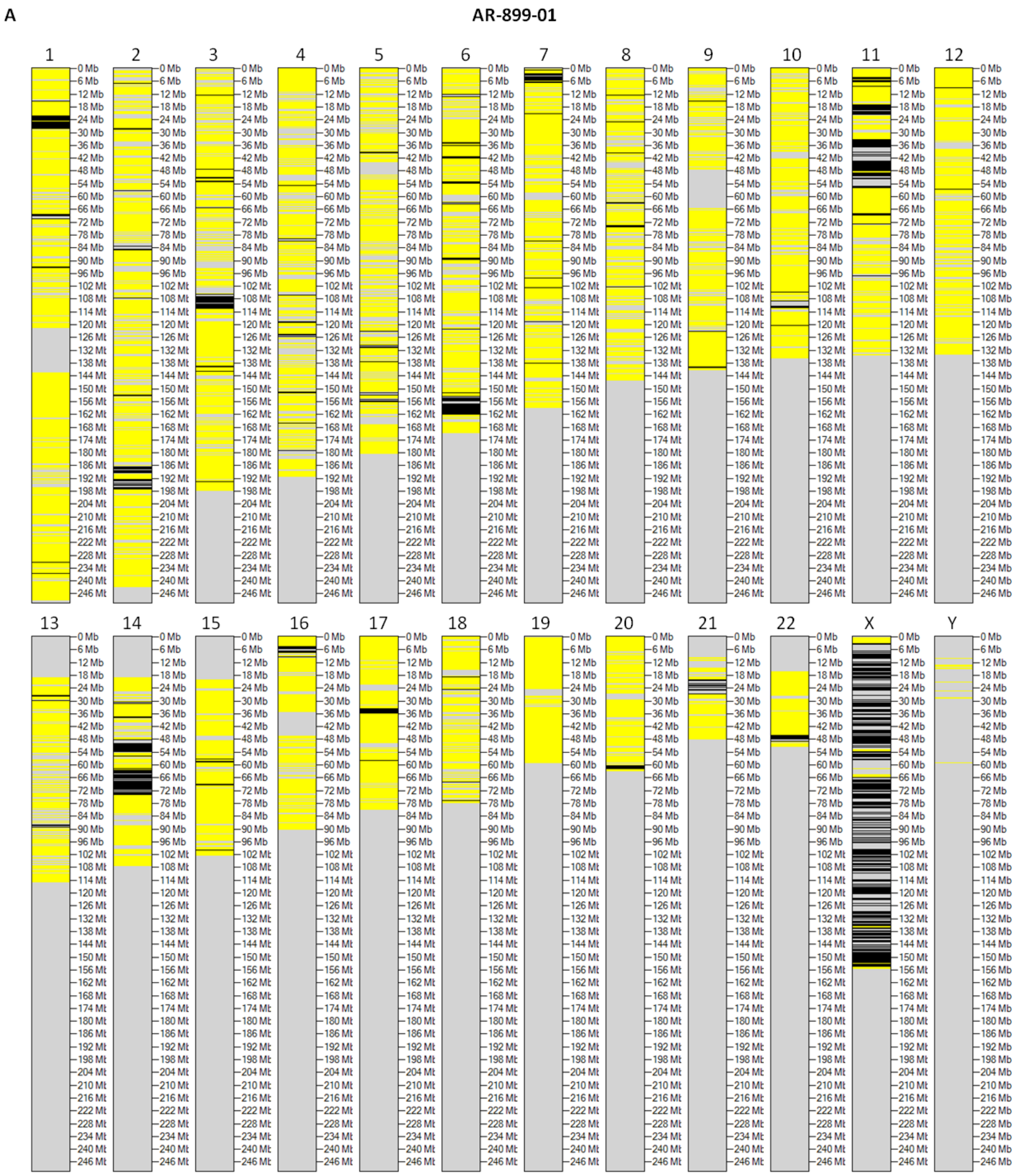

Figure S1. A single autozygous region segregates with Hutterite-type cataracts, confirming linkage to chromosome 6p22.2-p21.31. a-i. Regions of autozygosity (black) and heterozygosity (yellow) were determined from exome sequencing variant calls using AgileVariantMapper software.[1] A 9.5 Mb region on chromosome 6 that contains *MUC21* is the only span of absence

of heterozygosity (AOH) shared by all three exome-sequenced, affected individuals (**c, f, i**) and absent from their healthy parents (**a-b, d-e, g-h**).

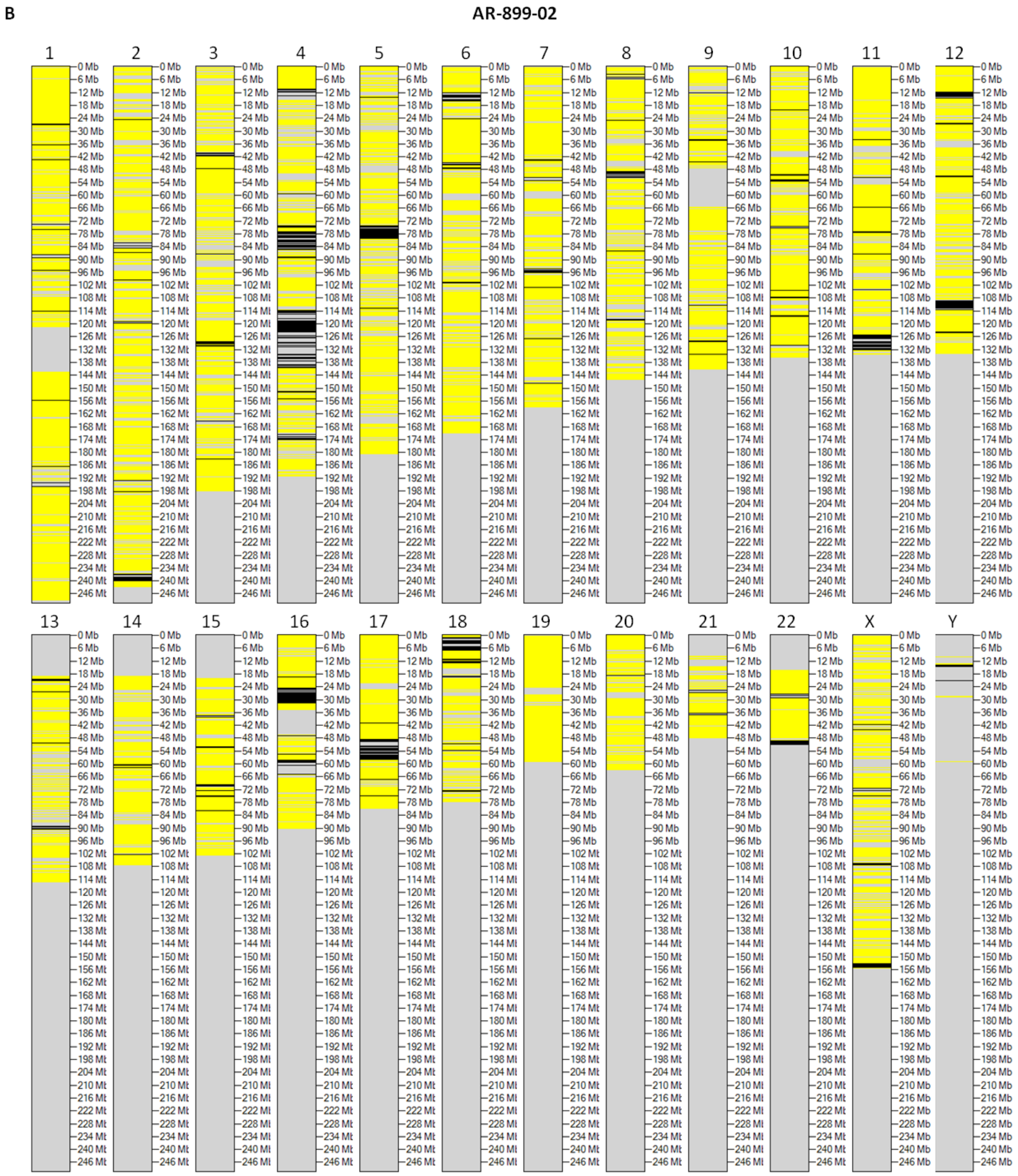

Figure S1, cont'd.

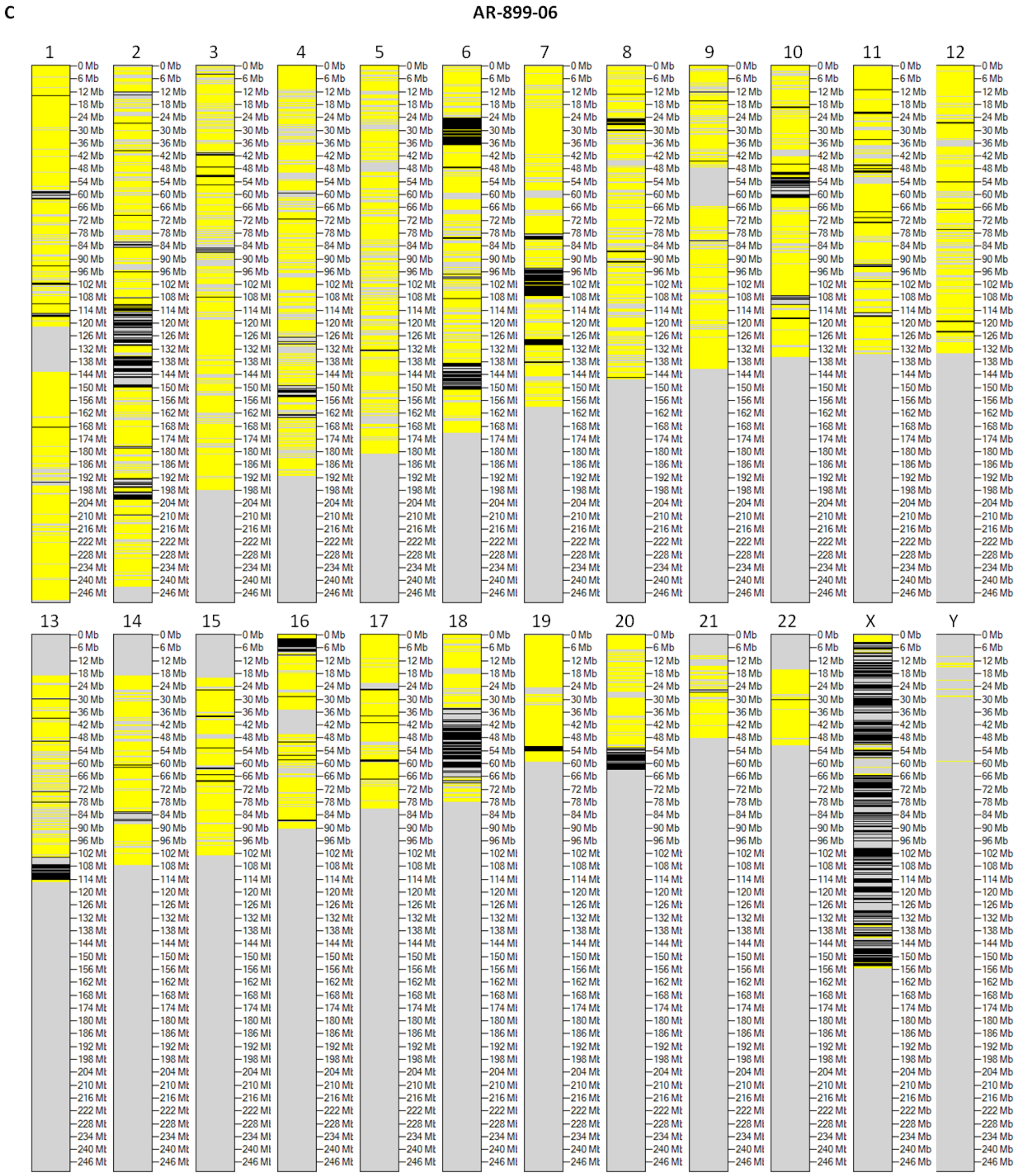

Figure S1, cont'd.

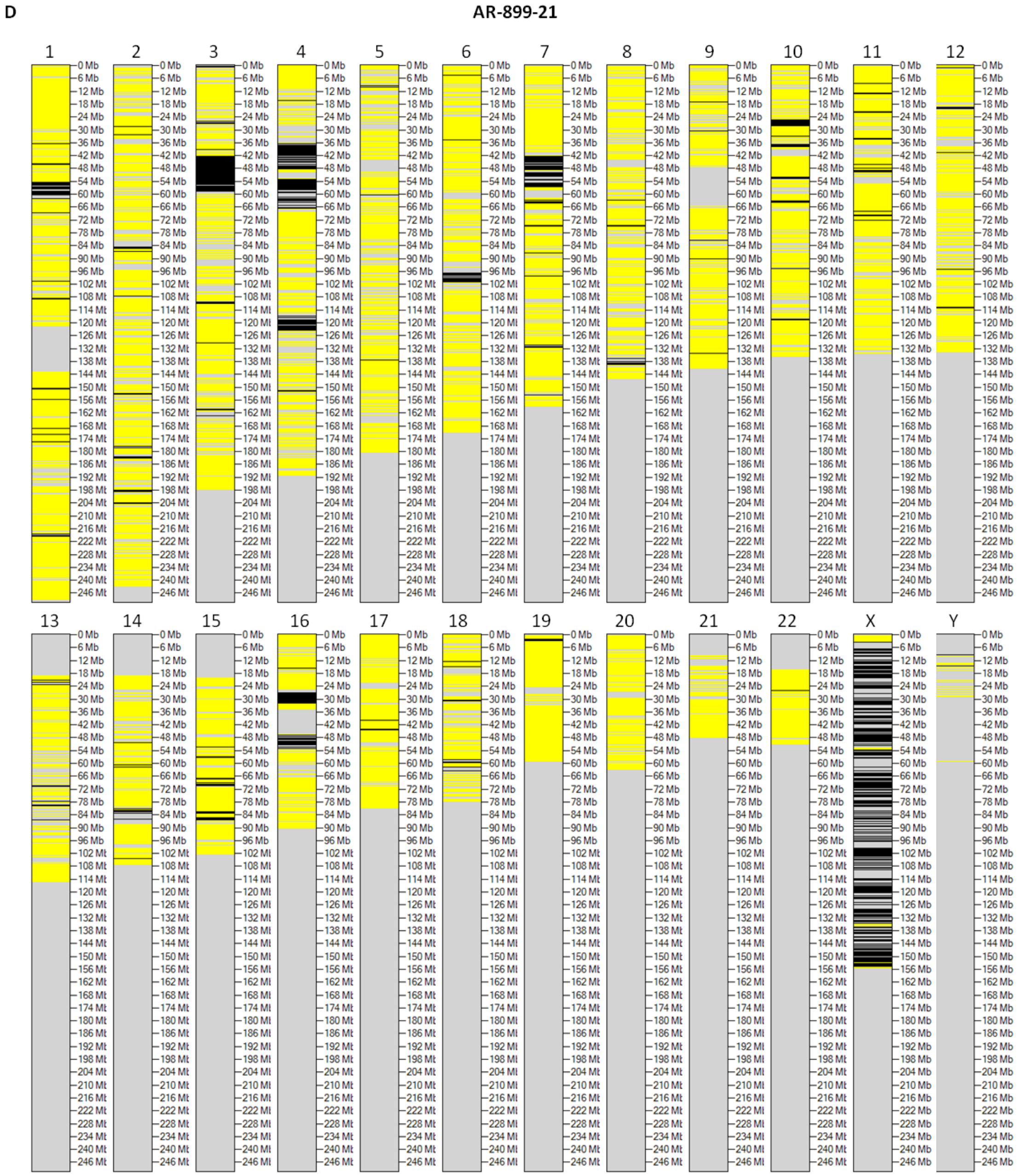

Figure S1, cont'd.

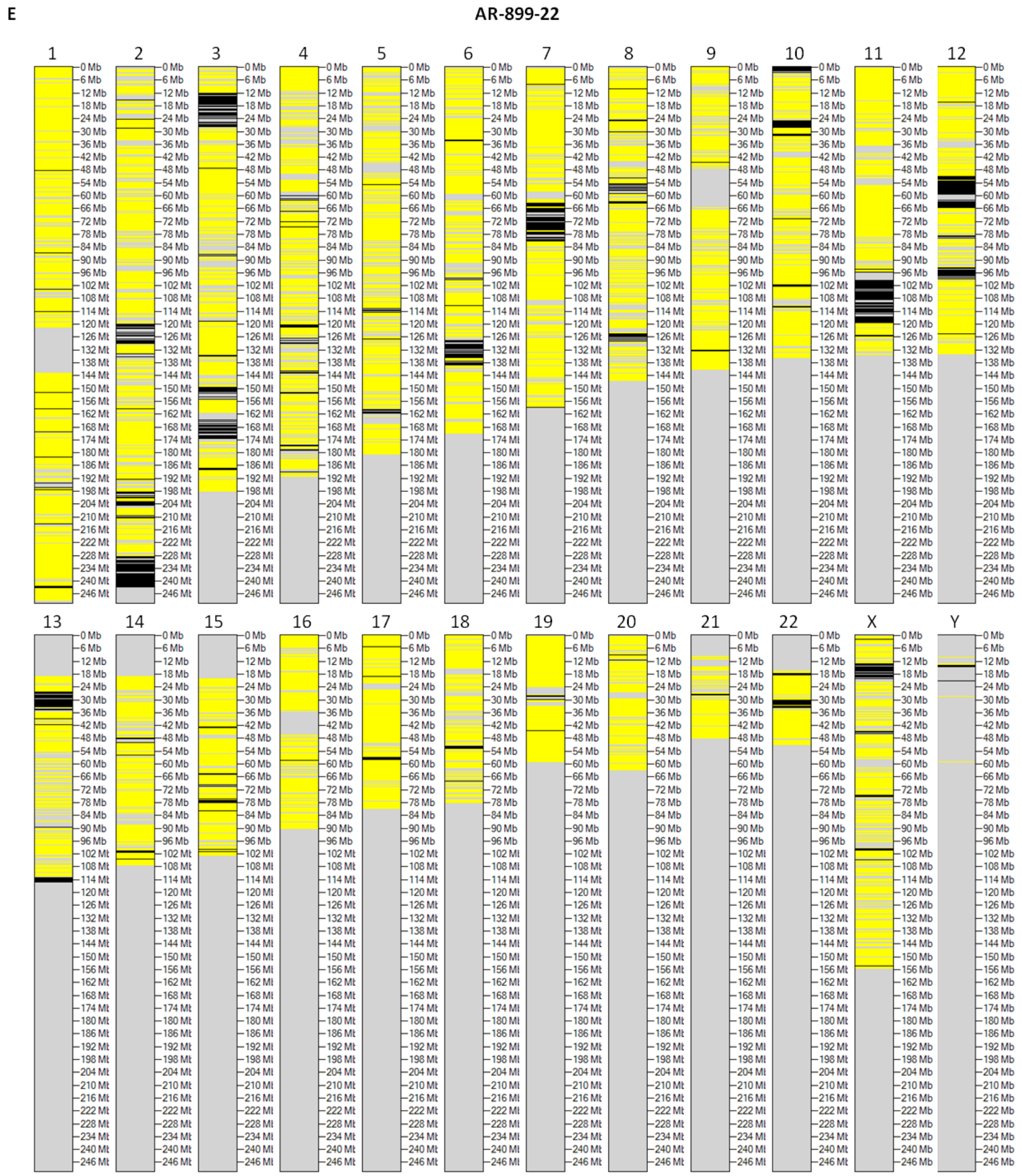

Figure S1, cont'd.

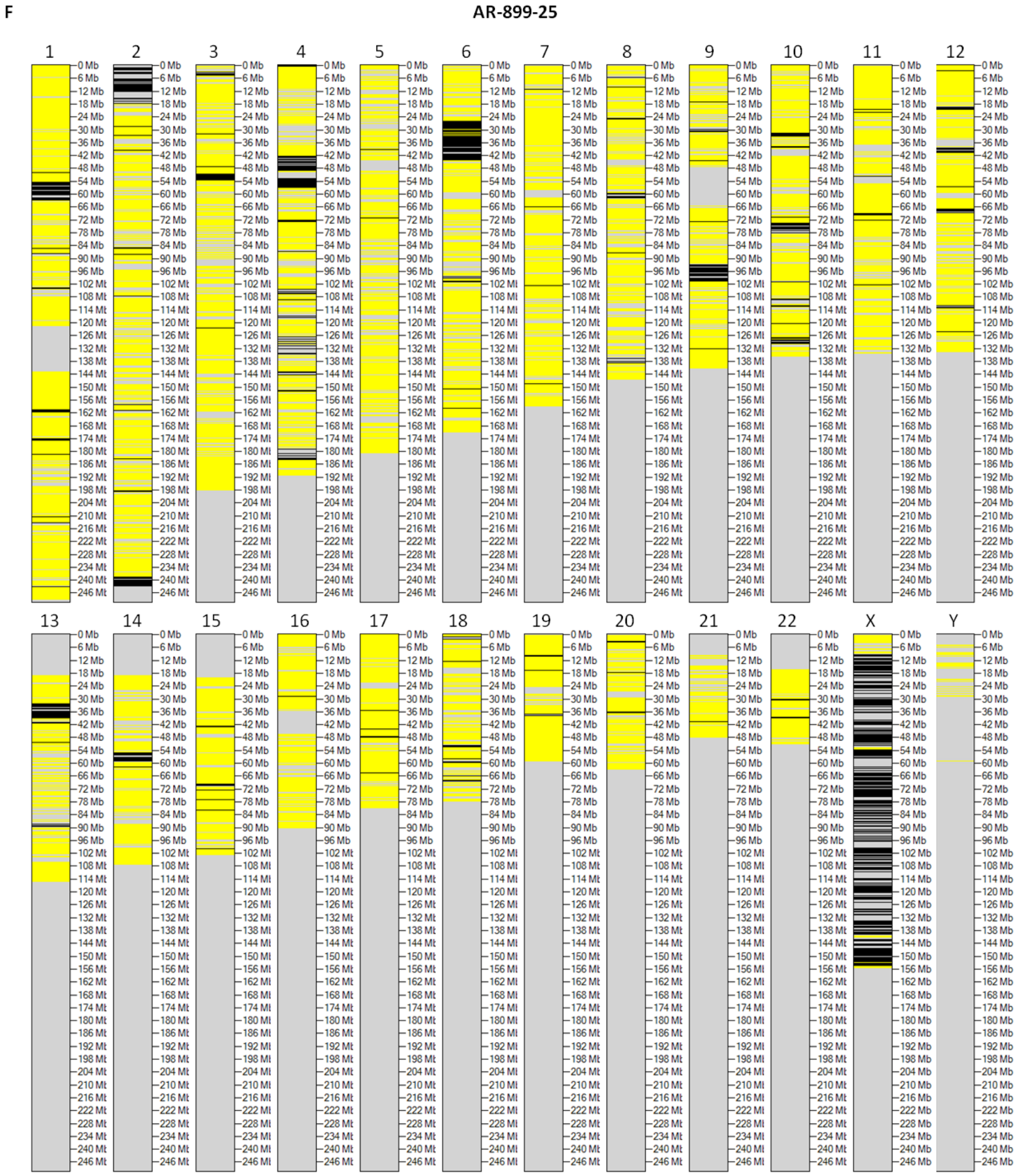

Figure S1, cont'd.

G

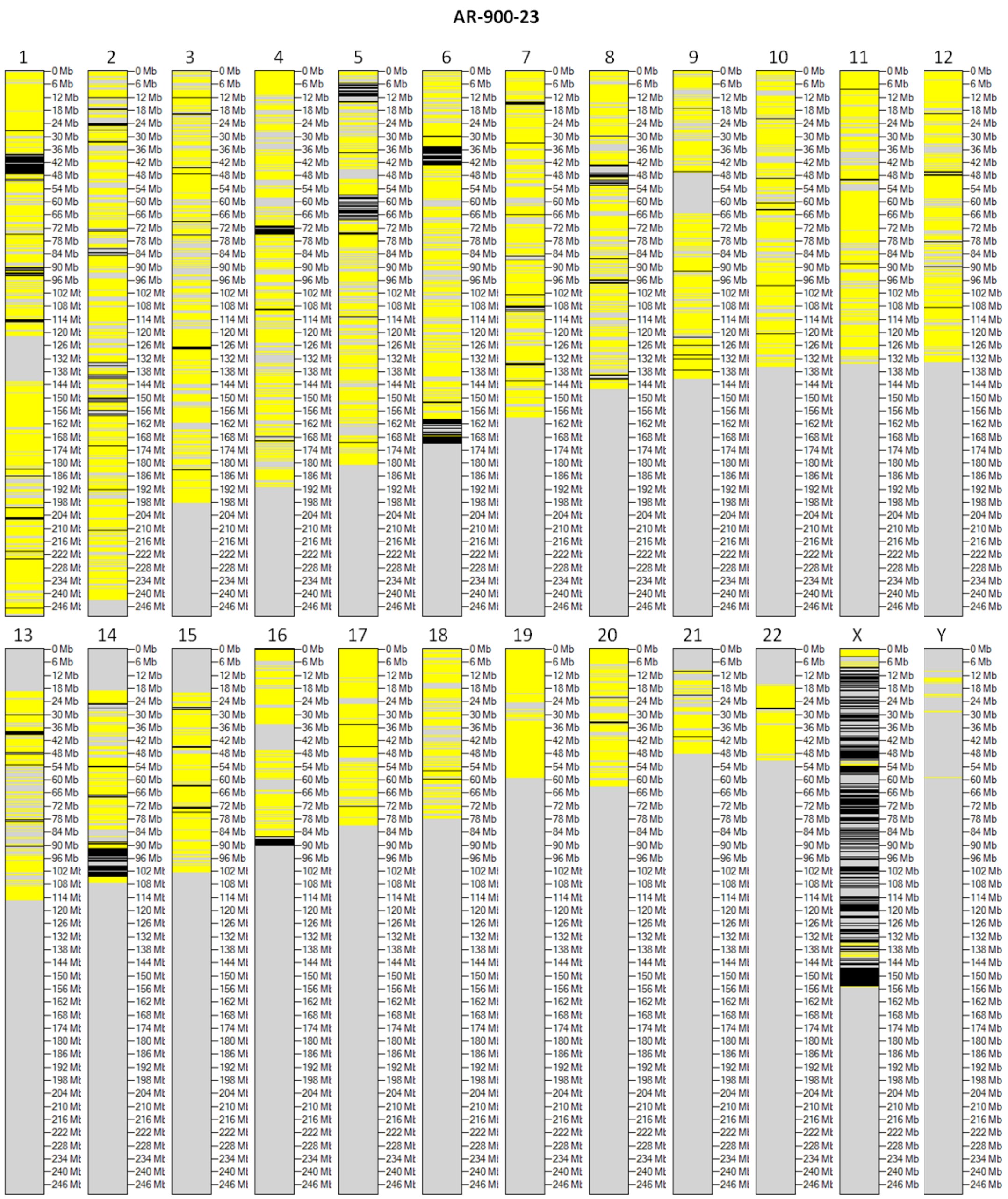

Figure S1, cont'd.

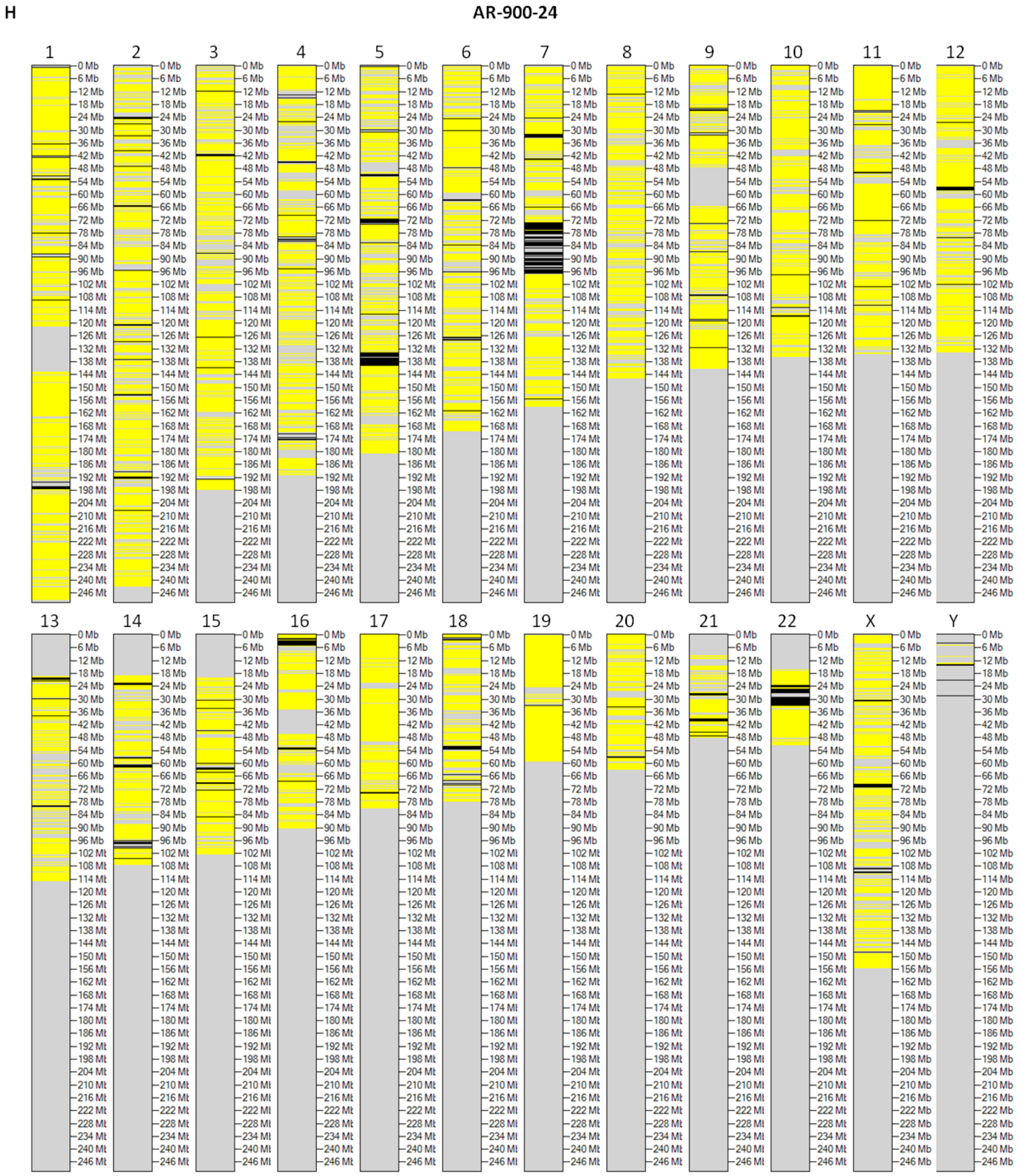

Figure S1, cont'd.

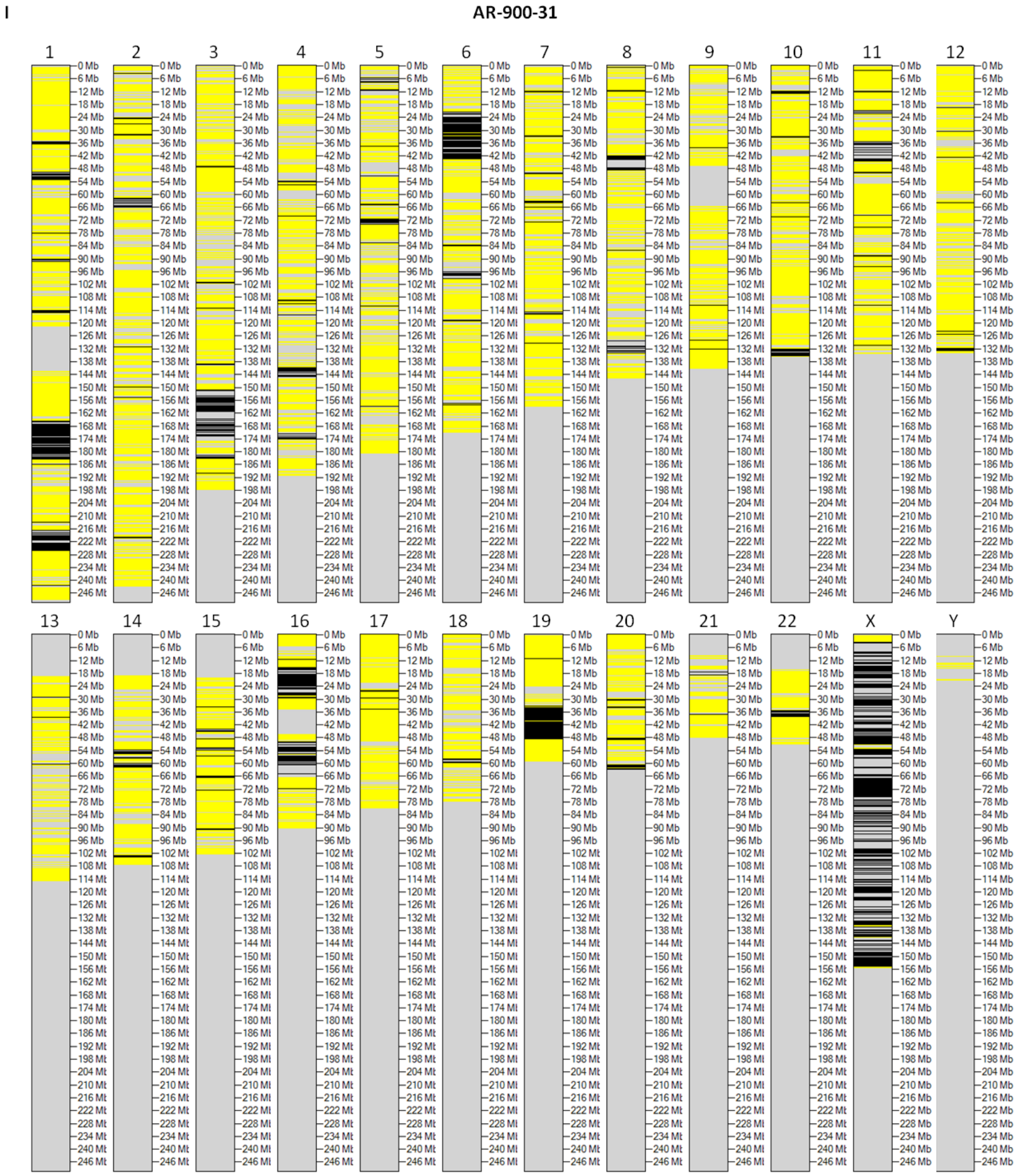

Figure S1, cont'd.

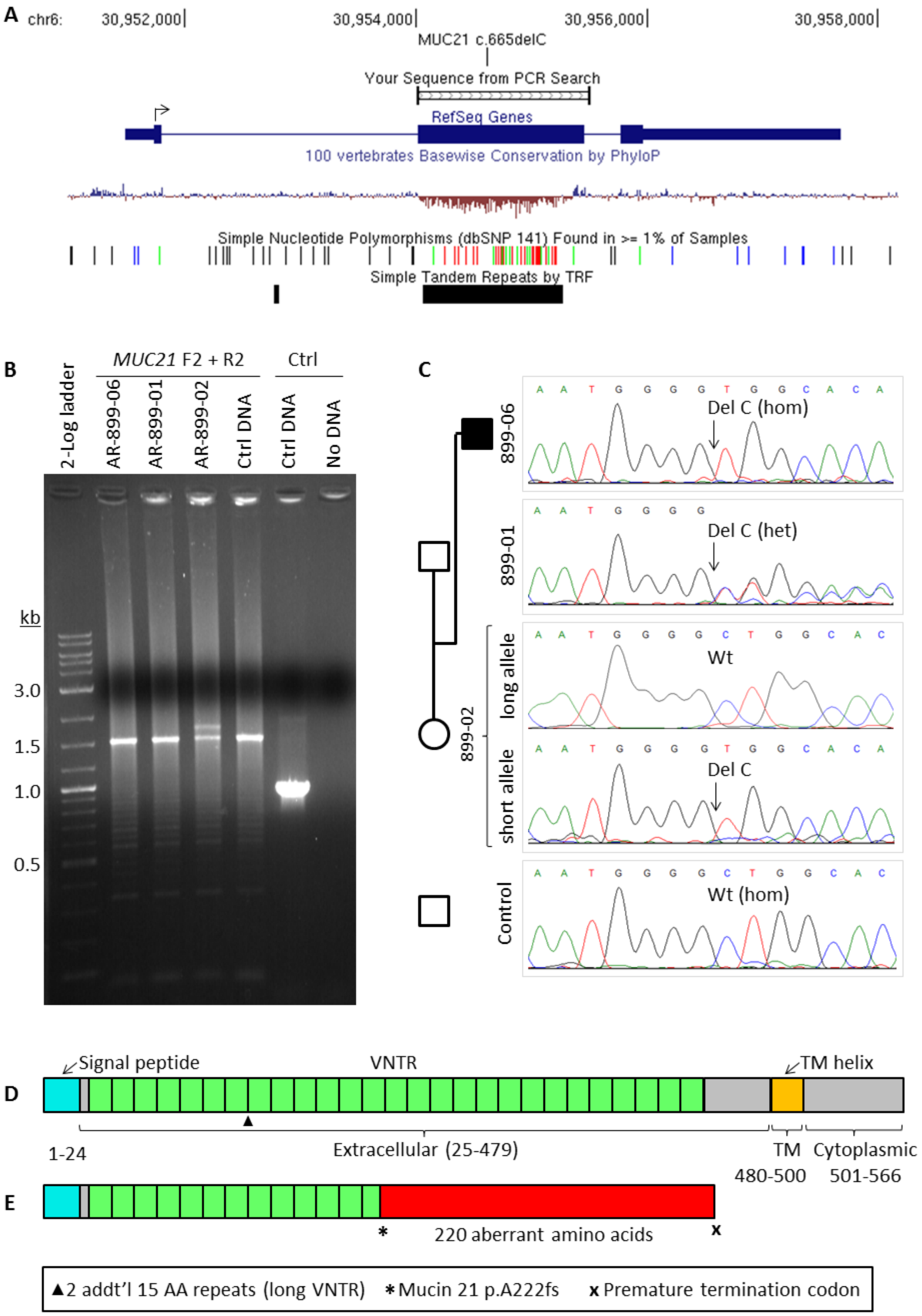

Figure S2. *MUC21* c.665delC is confirmed by Sanger sequencing and represents a predicted frameshift allele. a. *MUC21* c.665delC falls within a coding VNTR of 27 X 45 bp

repeats, necessitating a long (1,502 bp) PCR amplicon (“Your Sequence from PCR Search”) to sequence it. **b.** PCR of a trio provides substrate for sequencing and demonstrates that one amplicon in AR-899-02 is 90 bp longer than the other; this represents two additional 45 bp VNTR repeats (see Fig. S3). Note that all lanes show extraneous fast-moving bands with a periodicity of ~45 bp, necessitating gel-extraction of the primary amplicons prior to sequencing. **c.** Sanger sequencing of the *MUC21* c.665delC mutation in this trio confirms its presence and segregation with the phenotype. hom, homozygous; het, heterozygous. **d.** Wild type mucin 21. Number ranges signify amino acid positions. Domain information obtained from EBI Interpro (http://www.ebi.ac.uk/interpro/protein/Q5SSG8) and UniProt (http://www.uniprot.org/uniprot/Q5SSG8). AA, amino acid. **e.** Predicted effect of the *MUC21* c.665delC (p.A222Vfs*221) mutation, which introduces a frameshift in the thirteenth of 27 VNTR repeats. The mutation is predicted to introduce 220 aberrant amino acids before introducing a premature termination codon within exon 2 (see also Fig. S3). No specific evidence suggests escape from nonsense mediated decay (the premature termination codon is > 50-55 nucleotides upstream of the final exon-exon junction).[2]

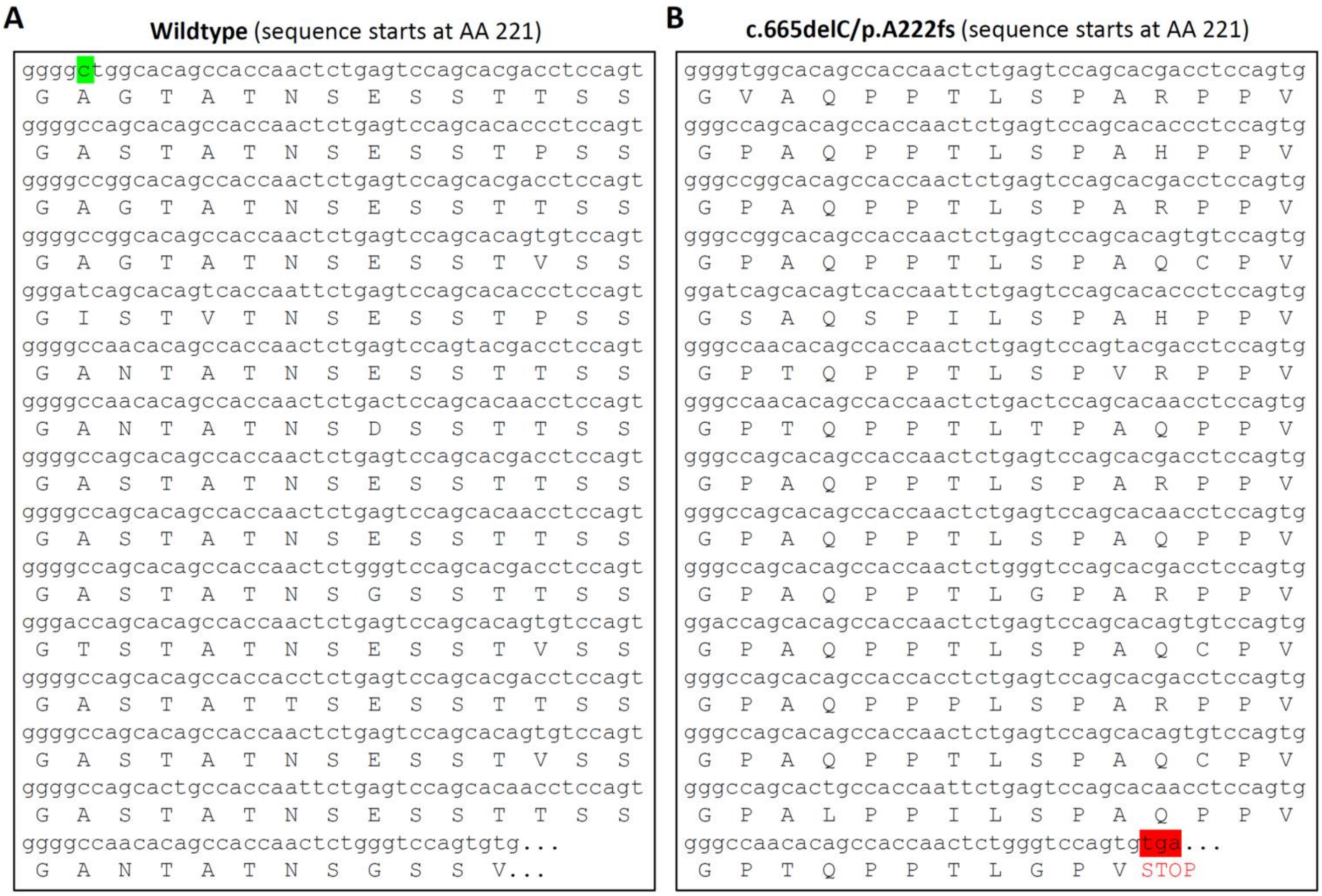

Figure S3. Predicted translational effect of *MUC21* c.665delC. The c.665delC mutation resides within the mucin domain-encoding portion of *MUC21*, composed of non-identical 45 bp (15 amino acid) repeats. **a.** Wild type sequence beginning with amino acid 221. The C base deleted in the mutant is shaded in green. 15 amino acids are shown per line, highlighting the mucin domain repeat structure. AA, amino acid. **b.** The mutation deletes a single C, altering amino acid 222 and the subsequent 219 amino acids. A premature stop codon (red) follows amino acid 441, just after the mucin repeats have ended.

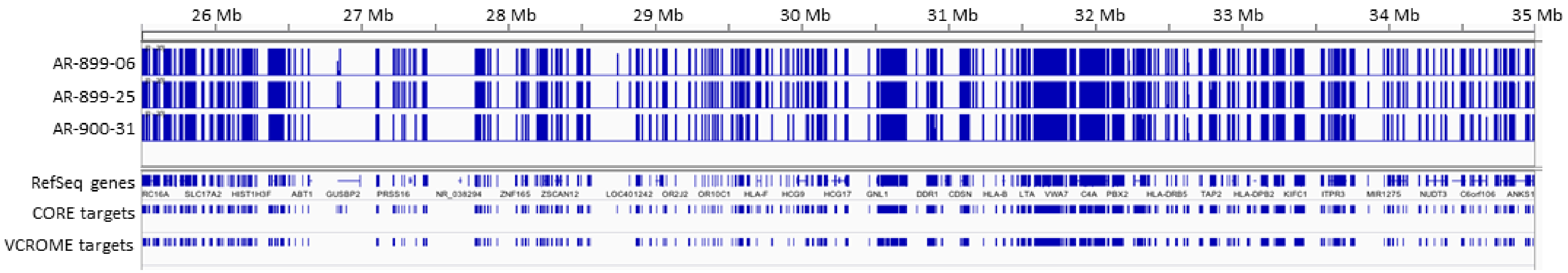

Figure S4. The vast majority of exons within the 9.5 Mb linked region are covered ≥ 20X. Top: Coverage over the 9.5 Mb chromosome 6 linked region in the three affected, exome sequenced individuals, with a y-axis limit of 20X. The vast majority of exons are covered ≥ 20X. **Bottom:** RefSeq genes, as well as targets of the CORE capture reagent (used for AR-899-06 and AR-899-25) and VCROME capture reagent (used for AR-900-31).

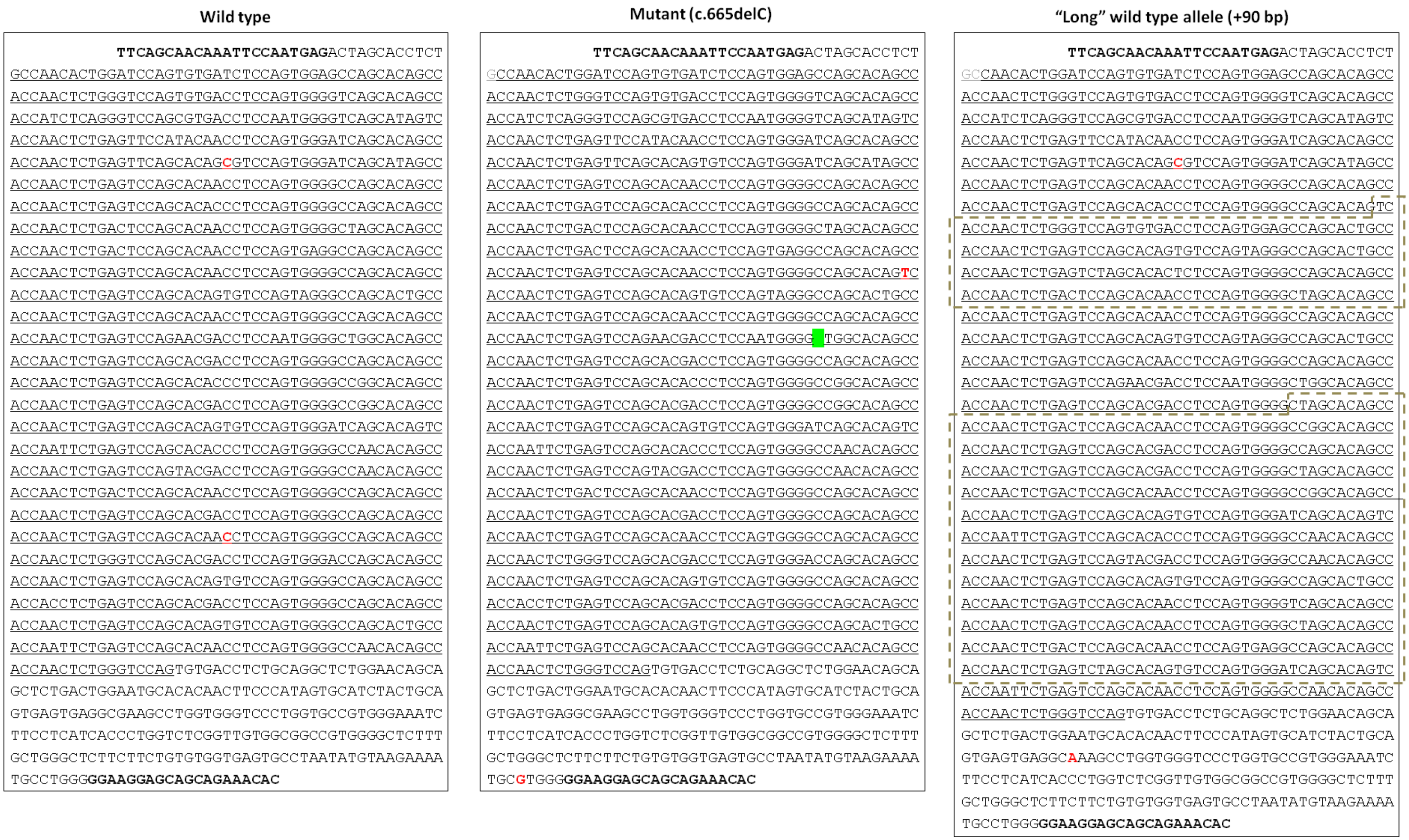

Figure S5. Complete sequences of *MUC21* exon 2. Sequences are derived from cloned PCR products and include the entire *MUC21* PCR amplicon. Forty-five nucleotides are shown per line, highlighting the mucin domain repeat structure (underlined bases). SNPs

shown in red text. *MUC21* primers shown in bold text. **a.** Sequence of wild type *MUC21* in AR-899-19, with 27 mucin repeats. The latter SNP is heterozygous. **b.** Sequence of the mutant (c.665delC) allele in AR-899-21. The deleted nucleotide is highlighted in green. **c.** Sequence of wild type allele in AR-899-21, with 29 (2 additional) mucin repeats. Mucin repeats outlined by dotted lines do not map well to the reference sequence; thus, no SNPs within them are indicated.

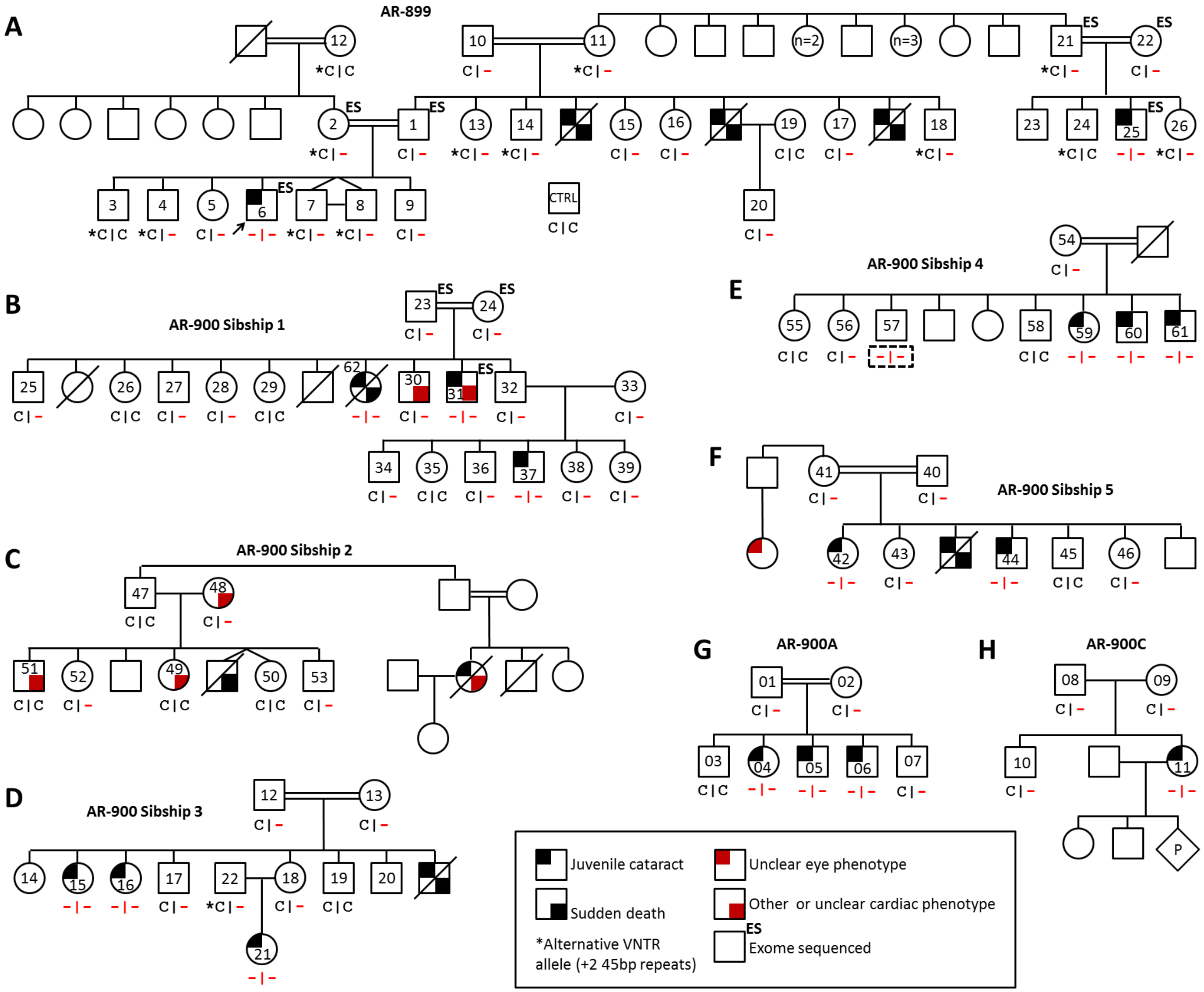

Figure S6. Hutterite-type cataracts co-segregate imperfectly with a homozygous *MUC21* variant (c.665delC). a, g, h. Families AR-899 (**a**), AR-900A (**g**), and AR-900C (**h**), and the majority of family AR-900 (**b-d, f**) demonstrate co-segregation of *MUC21* c.665delC (bold text directly below pedigree shapes; red text, mutant allele; black text, wt allele) with autosomal recessive cataracts. **e.** However, individual AR-900-57 (dotted line box) is homozygous for the mutant allele despite being phenotypically normal at age 47. Pedigrees have been trimmed to show only living generations/relevant individuals.

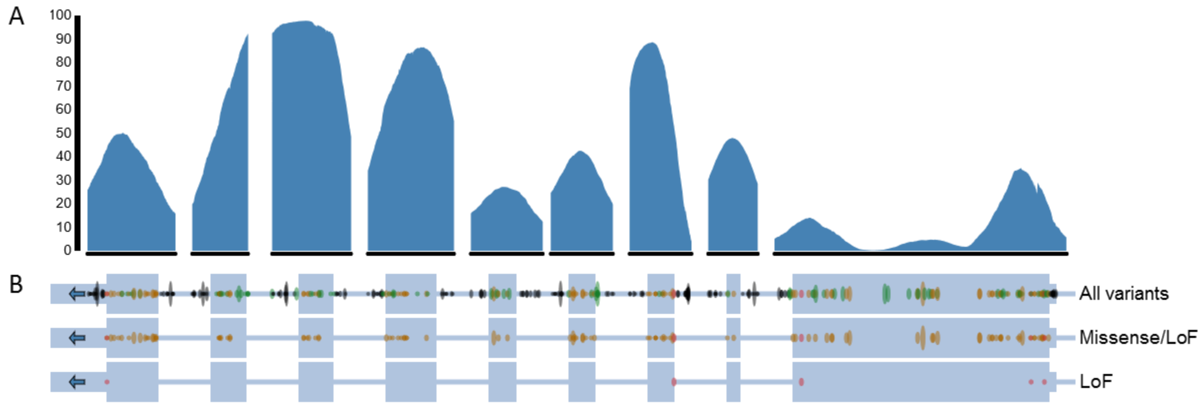

Figure S7. *LEMD2* harbors few loss-of-function alleles. a-b. Data reproduced from the Exome Aggregation Consortium (ExAC) browser[3] are plotted along the coding portion of the three exons of *MUC21*. **a.** Average sequencing read depth along the gene is low in some regions. **b.** While a number of *LEMD2* variants exist among the 60,706 ExAC exomes ("All variants"), loss of function variants ("LoF") are rare.

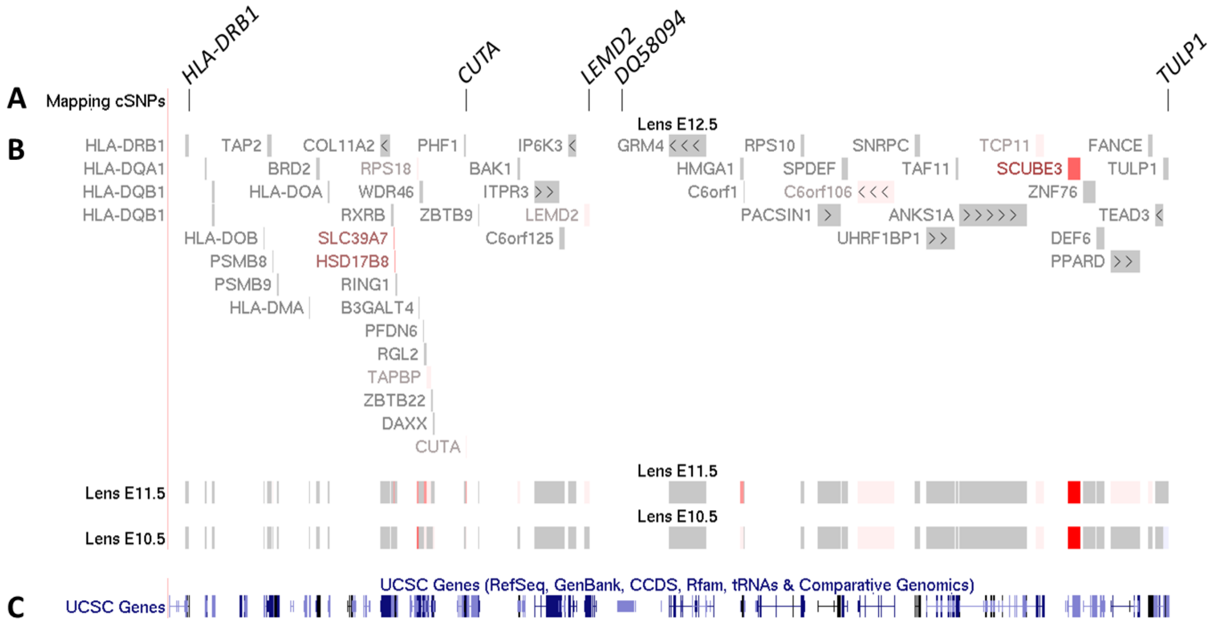

Figure S8. *LEMD2* is among the minority of genes within chromosome 6p21.32-p21.31 expressed in the developing mouse lens. a. Hutterite-type cataracts were mapped to a 0.5 Mb (between *CUTA* and *DQ580984* SNPs) to 2.9 Mb (between *HLA-DRB1* and *TULP1* SNPs) region (see Fig. 6). **b.** LEMD2 is among a minority of genes in this region expressed in the developing mouse lens (pink to red shading), and increases in expression from E10.5 to E12.5. Data from the iSYTE browser.[4] **c.** There are 81 RefSeq genes in maximum region and 13 in the minimum region.[5]

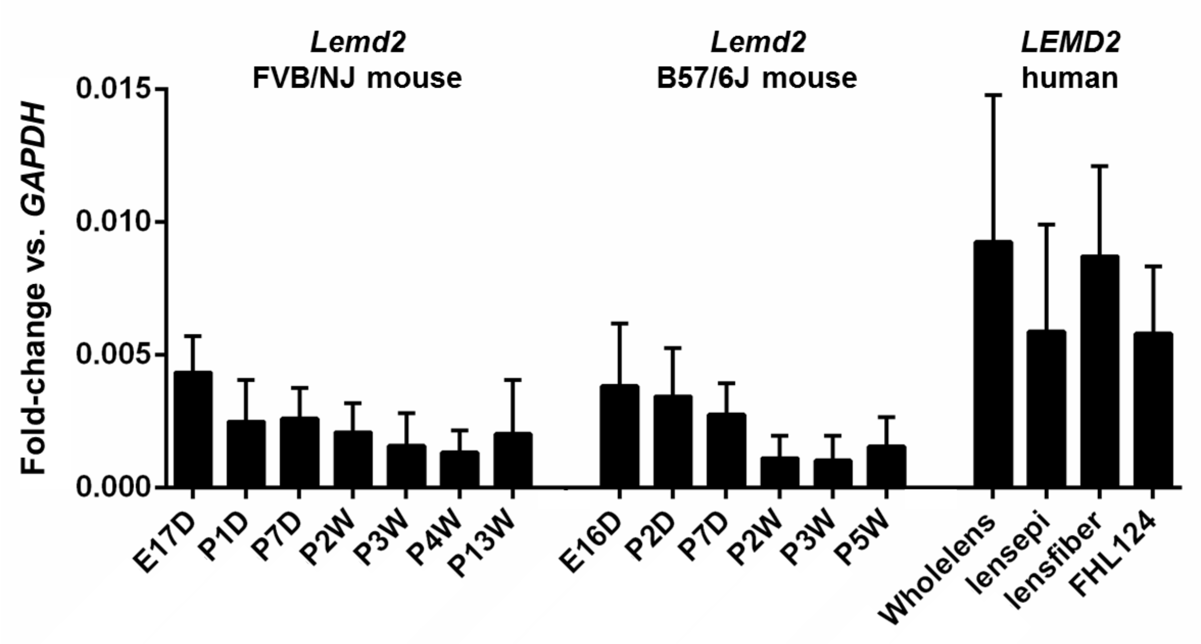

Figure S9. Expression of *LEMD2* and its murine ortholog, *Lemd2,* in the lens. Data are from quantitative real-time PCR, performed in quadruplicate. RT-PCR expression levels are plotted as $\Delta\Delta C_t$ displayed as fold-change from a *GAPDH* normalization control measured in 19-day postnatal chick lens. In both FVB/NJ mouse lens (**left** and B57/6J mouse lens (**center**), *Lemd2* expression is consistently detectable at multiple pre- and post-natal time points. *LEMD2* is expressed in human whole lens and two lens compartments (**right**), as well as in the FHL124 lens epithelium cell line. E, embryonic; P, postnatal; D, day; W, week. Error bars represent standard deviation.

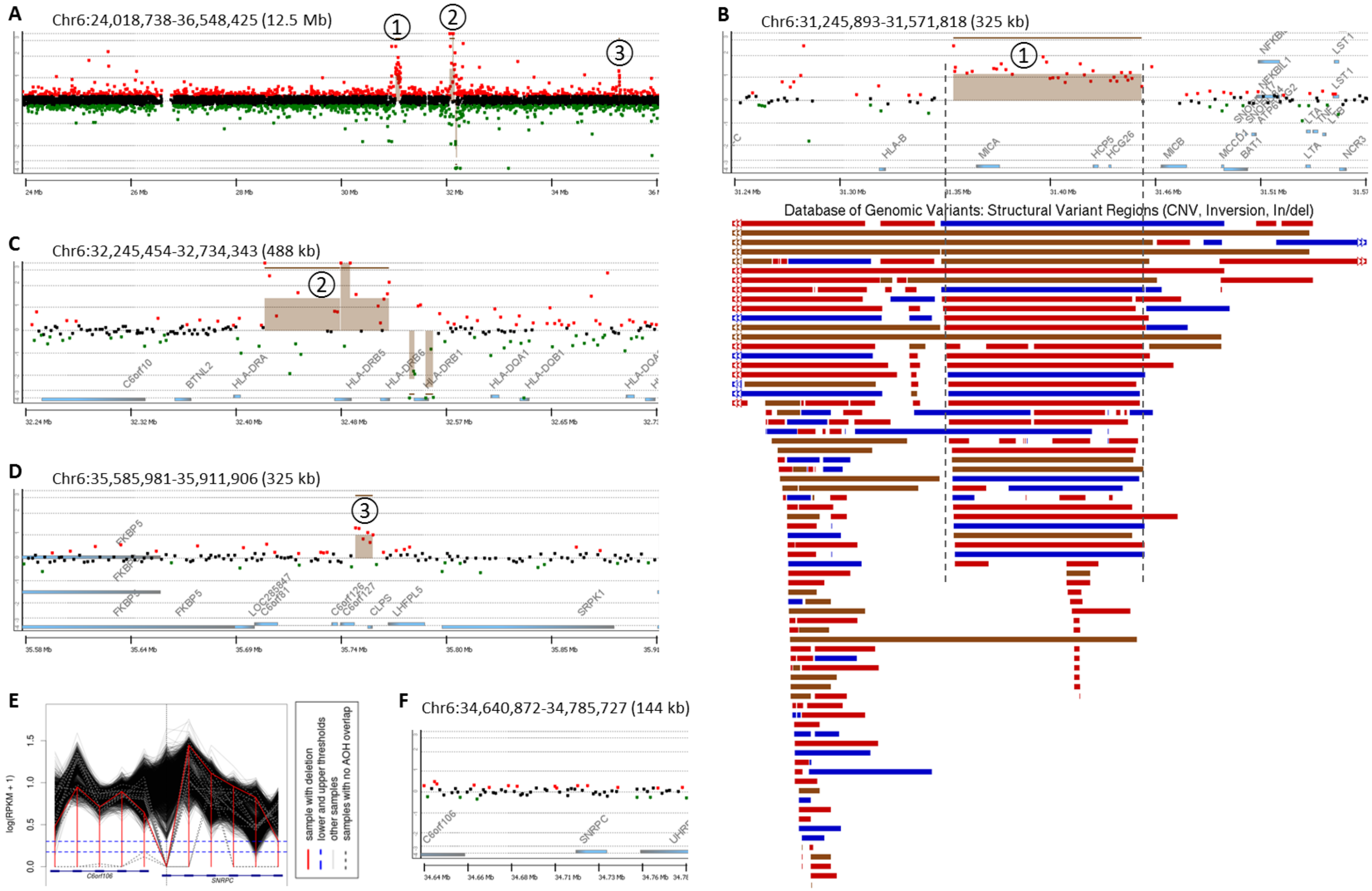

Figure S10. CNV analysis within the 9.5 Mb region linked to Hutterite cataract reveals no potential causative CNVs. a. A 1M probe genome-wide CGH array was run on a single affected individual, AR-899-06. In the region linked to Hutterite cataract

determined by autozygosity mapping, there are two potential CNVs (① and ②). One CNV just beyond this region (③) is also shown. **b.** CNV ① appears to be real (top) and is a homozygous duplication of a region commonly deleted and duplicated in healthy humans (bottom, “Database of Genomic Variants”) and flanked by low-copy repeats (not shown). It is outside of the narrow interval at 6p21.32-p21.31 linked to cataracts. **c.** CNV ② appears to be an artifact of the non-uniqueness of HLA genes in this region. **d.** CNV ③, which falls just outside of the linked region. **e.** Homozygous deletions were identified computationally from whole-exome sequencing data, revealing a potential single-exon deletion of *SNRPC* in AR-900-31. Read depth is indicated by the red line (AR-900-31) and black lines (4,123 other subjects). **f.** This potential CNV was not confirmed in AR-899-06 by array CGH.

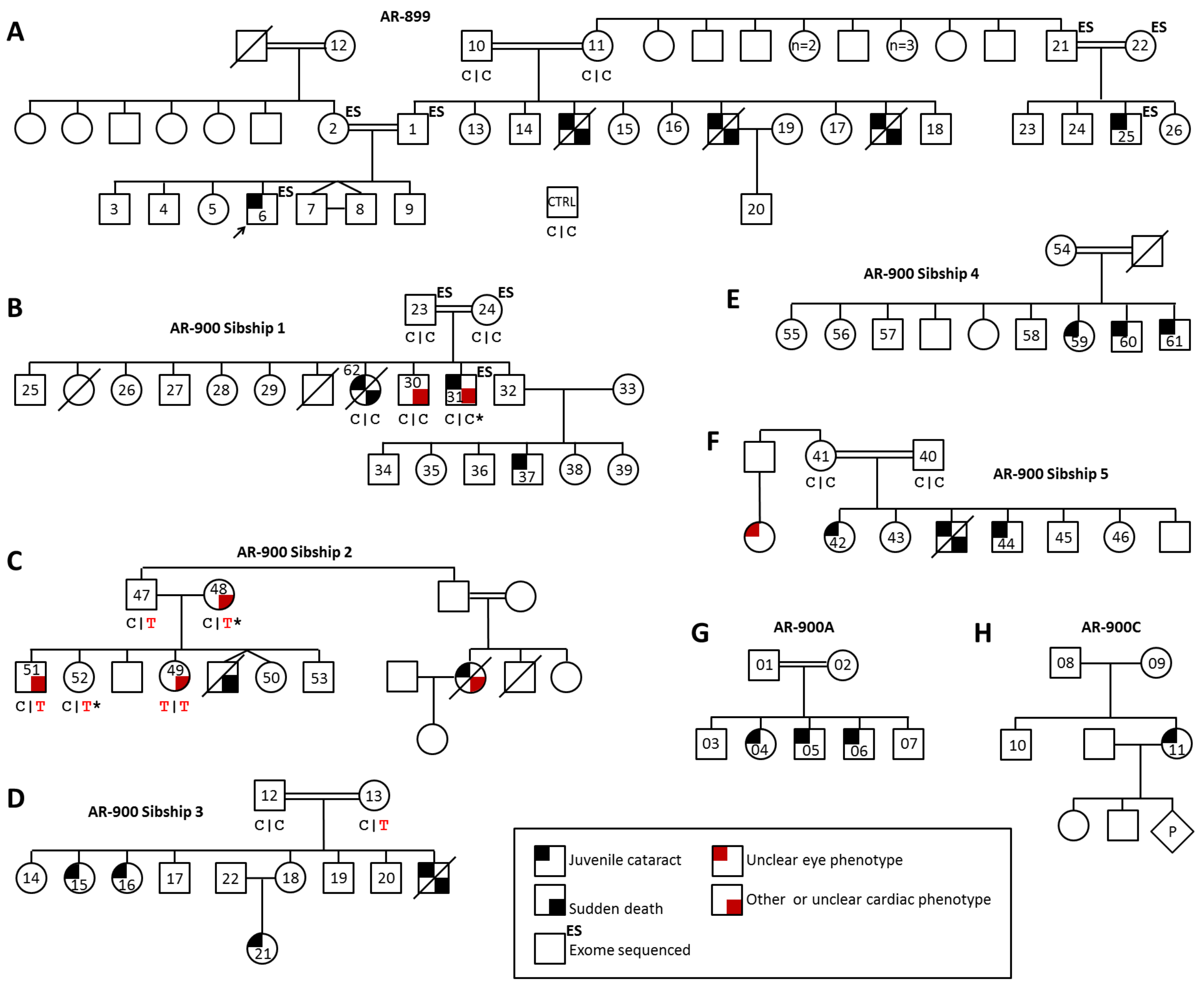

Figure S11. ***DSC2*** **c.C1660T does not segregate with sudden cardiac death in the majority of cataract sibships with sudden death. a-h.** Parents of individuals who died suddenly, as well as one individual experiencing sudden death (AR-900-62), were genotyped for the *DSC2* c.C1660T (p.Q554X) variant described by Gerull et al.[6] This variant is absent in obligate carrier parents in AR-899 and AR-900 sibships 1 and 5. Only in AR-900 sibship 2, as previously reported by Gerull et al.[6], are parents each heterozygous and a homozygous individual with a cardiac phenotype is found. AR-900 sibship 3 also contains a single carrier parent. Red text, mutant allele; black text, wt allele; *, variant confirmed by clinical testing by Gerull, et al. Pedigrees have been trimmed to show only living generations/relevant individuals.

Table S1. Characteristics of Hutterite-type cataracts in all reported individuals.

Pedigree No. Present Manuscript	Pedigree No. Shokeir and Lowry 1985	Pedigree No. Pearce et al. 1987.	Sex	First signs/ symptoms	Initial presentation	Second cataract mature, if time lag	Surgery and post-operative VA	Associated signs/symptoms	Negative signs/symptoms	Biochemical analyses
AR-900-31	V-9	VII-10	M	Infancy (looked at objects from closer distance than other children)	6y 0m (Mature cataract in one eye and early cataract in the other)	6y 3m (the milder cataract was found to have matured rapidly)	Mature cataract OS and posterior subcapsular opacity OD. Needling and aspiration OS. Three months later, R cataract was mature and operated on. Six months after initial presentation, a L retinal detachment was found which required two surgical procedures before reattachment occurred but which was complicated by a macular scar. 3 years after initial presentation, VA 20/25 R eye, counting fingers OS.	Slightly prominent frontal region, head circumference at 98th% (55cm)	Other growth parameters were on the 50th centile. Nl psychomotor development. Nl early growth and development and no visual problems. Exam 3 years after initial presentation showed nl intelligence and height.	No urinary reducing substances or amino acids in abnormal amounts, and RBC GALK activity was nl. Erythrocyte GALT was not tested.
AR-900-42	VI-1	VIII-1	F	Infancy (unusual appearance of pupils)	5y (OD anterior and posterior subcapsular opacities, granular polychromatophilic opacities in cortex; OS mature cataract, granular polychromatophilic opacities in anterior cortex)	-	Bilateral needling and aspiration. 20/50 OU, 20/25 with contact lenses.	Occasional nystagmus beginning in infancy	No hepatosplenomegaly. Nl hair, skin, teeth, nails, hearing, neurological exam. No growth abnormalities.	No urinary Gal or other reducing substances; RBC GALK and GALT (quantitative enzyme activity) nl; serum calcium, phosphorus, alkaline phosphatase, and glucose (both fasting and postprandial).
AR-900-59	VI-19 pedigree; VI-15 text	VII-28	F	?	5y (OD cataract; Clinical manifestation like that of younger brother (VI-16 in Shokeir and Lowry 1985))	Soon after first (OS anterior subscapular changes in cortex)	Both cataracts extracted. Corrected VA 20/25 OU. Nearly nl VA with correction.	-	No growth abnormalities. Nl intelligence and hearing and appropriate height, weight, and FOC for age. Neurologically nl.	No urinary reducing substances; RBC GALK activity nl.
AR-900-60	VI-20 pedigree; VI-16 text	VII-29	M	3y 8m (OS pupil turned white)	3y 10m (OS fairly mature cataract, VA LP; OD VA 20/40; Visual axes straight, pupillary responses nl, corneal diameters equal and nl, no sign of uveitis or perforation)	4y 2m (OD rapidly developing posterior cortical cataract, which quickly matured)	Staggered bilateral needling and aspiration. OS VA 20/40. Similar OD. “Later, a secondary membrane (remnants of the posterior capsule) was found to have developed in the pupillary area [of both eyes] and required repeated needling.” Corrected distance VA OD 20/20 and OS 20/25. IOP nl OU.	Allergies, penicillin sensitivity	Growth, development, general health, hair, nails, teeth nl. No myotonia. Height 50th centile, weight and FOC 75th%. No ocular trauma. Nl intelligence and hearing, appropriate weight. Neurologically nl.	Urine Gal and RBC GALK enzyme assay nl. Serum calcium, phosphorus, alkaline phosphatase, and blood glucose nl. No urinary reducing substances.

VA; visual acuity; Gal, galactose; GALK, galactokinase; GALT, galactose-1-phosphate uridyltransferase; RBC, red blood cell; Nl, normal; LP, light perception; OS, left eye; OD, right eye; OU, both eyes; - not performed or unknown. Reproduced in part from [7] and [8].

Table S1, cont'd.

Pedigree No. Present Manuscript	Pedigree No. Shokeir and Lowry 1985	Pedigree No. Pearce et al. 1987.	Sex	First signs/ symptoms	Initial presentation	Second cataract mature, if time lag	Surgery and post-operative VA	Associated signs/symptoms	Negative signs/symptoms	Biochemical analyses
AR-900-61	VI-21 pedigree; VI-17 text	VII-30	M	?	4y (OU mature cataracts)	-	Following surgery, secondary membranes developed OU requiring secondary membranectomy. No postoperative VA known.	-	No clinical or growth abnormalities. Nl intelligence and hearing and appropriate height, weight, and FOC for age. Neurologically nl.	No metabolic disorder. No urinary reducing substances; RBC GALK activity nl.
AR-900-62	V-8	VII-8	F	4y	4y 1m (OD mature cataract; OS early opacities)	4y 5m (OS mature) very dense, mature cataract(s)	Needling of both. Subsequent needlings of thickened posterior capsule were necessary. Postoperative VA 20/25 OU.	-	Nl early growth and development with no visual problems. At age 11y nl intelligence and height. Examination then showed no abnormalities apart from those related to eyes.	No urinary reducing substances or amino acids. RBC GALK activity nl. Quantitative determination of uronic acids were somewhat elevated, but results of GAG electrophoresis were nl. Erythrocyte GALT not tested.
AR-900, sibship 2	-	IX-1	F	?	3y 10m (First cataract mature)	3y 11m	Mature L cataract. R lens showed single anterior subcapsular vacuole but no opacities. L needling and aspiration. By five weeks after initial exam, R cataract was mature and surgerized. No postoperative VA known.	-	Nl growth and development, head circumference 75th centile.	-

VA; visual acuity; Gal, galactose; GALK, galactokinase; GALT, galactose-1-phosphate uridyltransferase; RBC, red blood cell; Nl, normal; LP, light perception; OS, left eye; OD, right eye; OU, both eyes; - not performed or unknown. Reproduced in part from [7] and [8].

Table S1, cont'd.

899-06	-	-	M	?	7y 0m (Referring optometrist noted "cortical spokes" in both eyes with a posterior subcapsular cataract OD). (At 7y 4m, ophthalmologist noted OD lens to have a mild degree of anterior subcapsular haze and a diffuse, white haze that extended from the posterior face of the lens anteriorly to approximately the central plane of the lens. This haze had an iridescent quality. OS lens showed a trace of diffuse haziness posteriorly. This was not the crystalline appearance of a typical posterior subcapsular cataract but rather a diffuse white haze in that area of the lens. There was no iridescence in this lens.)	7y 9m (OS lens appeared much as OD lens had been at the initial presentation. A mild, white anterior subcapsular haze was present with a much denser posterior haze – similar in appearance to OD lens 5 months previously though with no iridescence.	Cataract surgery and placement of an IOL. OD at 7y 4m; during aspiration of the lens it had the normal soft consistency typically encountered in a patient of this age. OS at 7y 9m.	R cornea showed a jagged scar infero-temporally that appeared to be nearly full thickness. There were no other signs of ocular trauma and the family and patient had no recollection of any injury to the eye.	-	-
AR-900-04	-	-	F	5y	?	?	?	-	-	-
AR-900-05	-	-	M	4y	?	?	?	-	-	-
AR-900-06	-	-	M	?	3y 7m (diffuse, white haziness in the posterior 1/3 to 1/2 of OD lens. OS lens was completely white.)	-	At the time of surgery both lenses were soft and aspirated easily.	-	-	-
AR-900-21	-	-	F	?	?	?	Cataract surgery at age 5.	-	-	-

VA; visual acuity; Gal, galactose; GALK, galactokinase; GALT, galactose-1-phosphate uridyltransferase; RBC, red blood cell; Nl, normal; LP, light perception; OS, left eye; OD, right eye; OU, both eyes; - not performed or unknown. Reproduced in part from [7] and [8].

Table S2. Primer sequences

LRRC16A F1	TTTGGGCTGCTGACAGATTT
LRRC16A R1	GCTTCTGGGAAGAAGGAAGG
HIST1H2AD F1	GACTCGCTCGGAGTAGTTGC
HIST1H2AD R1	TCCTCACTCTGCAGCTGGTA
HIST1H2BN F1	ACAAGGTGCTGAAGCAGGTC
HIST1H2BN R1	TGCCAGTTCTCTTTGGGAAG
OR2J3 F2	CCTCCTTCACCTTCTGGGTA
OR2J3 R2	TCTTCACTGCCCCTCTTACAA
TRIM40 F1	TTCAGGTAGACCACGGGAAC
TRIM40 R1	CCTCTTCTGTGCCCATTCTG
ABCF1 F1	TGCCTTCAGAATGTGAGGTG
ABCF1 R1	AGAGGGCAGGAAGAAAGGAG
MUC21 F2	TTCAGCAACAAATTCCAATGAG
MUC21 R2	GTGTTTCTGCTGCTCCTTCC
PSORS1C1 F1	CCGTTCACTTGACCCACTTT
PSORS1C1 R1	TTTGGGGTGATGAGGGATAC
HLA-C F1	CCCGGGAGATCTATAGGAGA
HLA-C R1	CCGGTTTCATTTTCGGTTTA
LY6G5C F1	GCAGCTTAGGAGGCTGAGAG
LY6G5C R1	GCAGTGGTAGGGTGTGAGGT
DDAH2 F1	ACTCTTCCAGCCAGCTCAGA
DDAH2 R1	CTGCATGTGATTCCAAGGTG
VWA7 F1	CCGGGAACCCTACAGTGAA
VWA7 R1	GAATGAGGGGAGCTCTTTCC
EHMT2 F1	GAAAACAGGCAAGCAAAAGG
EHMT2 R1	ACCGTCACCTTCCCTAAACA
HLA-DRB1 F1	CCTATTTTCCCCACCCCATA
HLA-DRB1 R1	AGACTTGCCTGCTTCTCTGG
CUTA F2	AGAGACCCAAAGACGGGATT
CUTA R2	AAGGGAGTGGGTTCTTTTGG
LEMD2 F1	GCTTGTTGCGGTAGACATCC
LEMD2 R2	TTAATCCCCCTTGGACCTTC
DQ580984 F1	GGCTTCTAGTTTGCGGTTCA
DQ580984 R1	GGCCTTCCGTCTCCATAGTA
TULP1 F1	CACGGGCTACCAGAAAGGT
TULP1 R1	GGAGATCCCTAGGGTGAGGA
DSC2 F1	CCCTGAGTGTAACCCTCCAA
DSC2 R1	CAGAGTGCATGTATCCAGCTT
CTRL F (SR)	TCCACAGGGTGACTGTGAAA
CTRL R (SR)	TAGGGACACCTGGGAATTTG
M13 forward	GTAAAACGACGGCCAG
M13 reverse	GTAAAACGACGGCCAG
Lemd2 forward (RT-PCR)	GTGGGTGAAGATGGGCAAG
Lemd2 reverse (RT-PCR)	GTGCAGCAGCTCCAACAG
Gapdh forward (RT-PCR)	CGTCCCGTAGACAAAATGGT
Gapdh reverse (RT-PCR)	TCAATGAAGGGGTCGTTGAT
LEMD2 forward (RT-PCR)	CTGTCAGGCCAAGCAGAAG
LEMD2 reverse (RT-PCR)	GGCTTCCATAACAGGAATGC
GAPDH forward (RT-PCR)	AGGGCTGCTTTTAACTCTGGT
GAPDH reverse (RT-PCR)	GACAAGCTTCCCGTTCTCAG

Table S3. cSNPs used to fine-map the cataract locus within 6p22.2-p21.31.

**No.**	**Position (hg19)**	**Ref**	**Var**	**Gene**	**dbSNP142**	**dbSNP allele freq[1] (via UCSC)**	**ExAC allele freq[1]**	**ExAC homozygotes[1]**
1	chr6:25420350	A	C	*LRRC16A*	rs41271807	0.11541	0.1486	1499
2	chr6:26199454	C	T	*HIST1H2AD*	rs41266803	0.05511	0.05406	176
3	chr6:27806827	C	T	*HIST1H2BN*	rs112586220	0.00280	0.01588	35
4	chr6:29080344	C	A	*OR2J3*	rs3749977	0.18580	0.2972	5879
5	chr6:30114955	C	T	*TRIM40*	rs757262	0.08896	0.2100	3061
6	chr6:30558125	C	T	*ABCF1*	-	-	-	-
7	chr6:30954617	C	del	***MUC21***	-	-	-	-
8	chr6:31106489	G	A	*PSORS1C1*	rs1265096	0.00849	0.05191	309
9	chr6:31239050	G	T	*HLA-C*	rs713032	0.23822	0.1410	1524
10	chr6:31647001	A	G	*LY6G5C*	rs11575852	0.00948	0.02715	67
11	chr6:31695368	G	A	*DDAH2*	rs11540321	0.01038	0.03238	129
12	chr6:31737818	C	G	*VWA7*	rs115526889	0.00399	0.01896	42
13	chr6:31864538	G	A	*EHMT2*	rs115884658	0.00280	0.01811	45
14	chr6:32557483	C	T	*HLA-DRB1*	rs9270303	0.22085	0.2317	?
15	chr6:33384473	C	T	*CUTA*	rs41267649	0.00559	0.02032	57
16	Chr6:33756856	A	C	***LEMD2***	-	-	-	-
17	chr6:33851052	C	A	*DQ580984*	rs11759059	0.13119	-	-
18	chr6:35479574	G	C	*TULP1*	rs7764472	0.87440	0.8602	14180

[1]As of 16 Mar 2015

Table S4. Loss of Function Alleles in *LEMD2* in the Exome Aggregation Consortium (ExAC) Browser

Variant	Chr	Position	Protein Consequence	Filter	Annotation	Allele Count	Allele Number	Number of Homozygotes	Allele Frequency
6:33740410 G / A	6	33740410	p.Arg503Ter	PASS	stop gained	1	119494	0	0.000008369
6:33752205 C / A	6	33752205		PASS	splice acceptor	7	120066	0	0.00005830
6:33756184 G / C	6	33756184	p.Ser237Ter	PASS	stop gained	1	22918	0	0.00004363
6:33756842 AG / A	6	33756842	p.Phe18SerfsTer98	PASS	frameshift	1	102828	0	0.000009725
6:33756880 G / T	6	33756880	p.Ser5Ter	PASS	stop gained	1	92256	0	0.00001084
								**Total:**	**0.000130864**

Data reproduced from [3].

Table S5. Genes within the 2.9 Mb linked region that are expressed in mouse lens (data from Hoang et al.[9]).

Start (Chr6)	Stop (Chr6)	Gene
32578769	32589836	HLA-DRB1
32637392	32654685	HLA-DQA1
32659464	32666689	HLA-DQB1
32706144	32706803	MTCO3P1
32741386	32746887	HLA-DQA2
32749912	32749979	MIR3135B
32756098	32763553	HLA-DQB2
32812763	32817048	HLA-DOB
32821833	32838770	TAP2
32840717	32844935	PSMB8
32844086	32846500	PSMB8-AS1
32845209	32853971	TAP1
32854161	32859851	PSMB9
32876478	32880074	PPP1R2P1
32894176	32903758	LOC100294145
32896402	32896489	HLA-Z
32934629	32941070	HLA-DMB
32948614	32953122	HLA-DMA
32968660	32981505	BRD2
33004182	33009612	HLA-DOA
33064569	33080778	HLA-DPA1
33075926	33089696	HLA-DPB1
33079299	33080011	RPL32P1
33091482	33093314	HLA-DPA2
33103693	33107144	COL11A2P1
33112516	33129113	HLA-DPB2
33131197	33131343	HLA-DPA3
33162692	33192468	COL11A2
33193585	33200696	RXRB
33199601	33199692	RNY4P10
33200826	33204437	SLC39A7
33204637	33206831	HSD17B8
33207835	33207944	MIR219A1
33208509	33212722	RING1
33215574	33216183	ZNF70P1
33249536	33254890	HCG25
33250272	33271965	VPS52
33272075	33276504	RPS18
33277140	33278825	B3GALT4
33278905	33289527	WDR46
33287227	33287289	MIR6873
33289597	33290934	PFDN6
33290245	33290325	MIR6834
33291654	33299460	RGL2
33299694	33314387	TAPBP
33314405	33317942	ZBTB22
33318558	33323016	DAXX
33323628	33324116	LOC102723452
33338978	33339495	MYL8P
33364724	33366362	LYPLA2P1
33389356	33389479	RPL35AP4

Yellow, expressed. Grey, not expressed.

Table S5, cont'd.

33391536	33409924	KIFC1
33400015	33400644	RPL12P1
33410996	33416453	PHF1
33416542	33418288	CUTA
33420070	33453689	SYNGAP1
33438331	33438437	MIR5004
33454579	33457544	ZBTB9
33572546	33580296	BAK1
33583699	33589026	GGNBP1
33586106	33593338	LINC00336
33621379	33696574	ITPR3
33631244	33633636	LOC101929188
33696761	33711751	UQCC2
33698128	33698234	MIR3934
33721638	33746985	IP6K3
33771213	33789129	LEMD2
33794672	33804016	MLN
33889511	33896907	LINC01016
33899135	33899200	MIR7159
33911102	33918208	LOC100653005
33999972	34000051	MIR1275
34018643	34155622	GRM4
34189732	34191141	KRT18P9
34219372	34220937	CYCSP55
34236800	34246231	HMGA1
34240673	34240736	MIR6835
34246380	34249108	C6orf1
34263270	34263723	RPL35P2
34277591	34284549	LOC102723521
34287194	34392680	NUDT3
34287194	34426125	RPS10-NUDT3
34417454	34426125	RPS10
34466061	34535223	PACSIN1
34537802	34556333	SPDEF
34576101	34576757	LOC100101247
34587280	34696850	C6orf106
34616522	34617324	RPL7P25
34696944	34697470	LOC101929243
34744140	34744718	RPS10P13
34757094	34773857	SNRPC
34792017	34880152	UHRF1BP1
34877778	34888071	TAF11
34889261	35091413	ANKS1A
35118071	35141641	TCP11
35150678	35163658	LOC101929263
35214047	35253079	SCUBE3
35228299	35259444	LOC101929285
35259488	35295985	ZNF76
35297818	35321771	DEF6
35342558	35428191	PPARD
35446210	35449865	MKRN6P
35452339	35467141	FANCE
35468401	35470781	RPL10A
35470508	35470579	MIR7111
35473597	35497084	TEAD3
35497874	35512902	TULP1

Yellow, expressed. Grey, not expressed.

References:

1. Carr IM, Bhaskar S, O' Sullivan J, et al. Autozygosity mapping with exome sequence data. Hum Mutat 2013;34(1):50-6.

2. Miller JN, Pearce DA. Nonsense-mediated decay in genetic disease: friend or foe? Mutat Res Rev Mutat Res 2014;762:52-64.

3. Exome Aggregation Consortium. Exome Aggregation Consortium (ExAC) Browser. 2014.

4. Lachke SA, Ho JW, Kryukov GV, et al. iSyTE: integrated Systems Tool for Eye gene discovery. Invest Ophthalmol Vis Sci 2012;53(3):1617-27.

5. OMIM. Online Mendelian Inheritance in Man.

6. Gerull B, Kirchner F, Chong JX, et al. Homozygous founder mutation in desmocollin-2 (*DSC2*) causes arrhythmogenic cardiomyopathy in the Hutterite population. Circ Cardiovasc Genet 2013;6(4):327-36.

7. Shokeir MHK, Lowry RB. Juvenile cataract in Hutterites. Am J Med Genet 1985;22(3):495-500.

8. Pearce WG, Mackay JA, Holmes TM, et al. Autosomal recessive juvenile cataract in Hutterites. Ophthalmic Paediatr Genet 1987;8(2):119-24.

9. Hoang TV, Kumar PKR, Sutharzan S, et al. Comparative transcriptome analysis of epithelial and fiber cells in newborn mouse lenses with RNA sequencing. Mol Vis 2014;20:1491-517.
